# Targeted *in silico* characterization of fusion transcripts in tumor and normal tissues via FusionInspector

**DOI:** 10.1016/j.crmeth.2023.100467

**Published:** 2023-05-08

**Authors:** Brian J. Haas, Alexander Dobin, Mahmoud Ghandi, Anne Van Arsdale, Timothy Tickle, James T. Robinson, Riaz Gillani, Simon Kasif, Aviv Regev

**Affiliations:** 1 Klarman Cell Observatory, Broad Institute of MIT and Harvard, Cambridge, MA, USA; 2 Graduate Program in Bioinformatics, Boston University, Boston, MA 02215, USA; 3 Cold Spring Harbor Laboratory, New York, NY 11724, USA; 4 Monte Rosa Therapeutics, Boston, MA 02210, USA; 5 Department of Obstetrics and Gynecology and Women’s Health, Albert Einstein Montefiore Medical Center, Bronx, NY 10461, USA; 6 Department of Genetics, Albert Einstein College of Medicine, Bronx, NY 10461, USA; 7 School of Medicine, University of California San Diego, La Jolla, CA 92093, USA; 8 Department of Pediatric Oncology, Dana-Farber Cancer Institute, Boston, MA 02215, USA; 9 Cancer Program, Broad Institute of Harvard and MIT, Cambridge, MA 02142, USA; 10 Department of Pediatrics, Harvard Medical School, Boston, MA 02215, USA; 11 Boston Children’s Hospital, Boston, MA 02115, USA; 12 Department of Biomedical Engineering, Boston University, Boston, MA 02215, USA; 13 Department of Biology, Massachusetts Institute of Technology, Cambridge, MA 02139, USA; 14 Howard Hughes Medical Institute, Chevy Chase, MD 20815, USA

**Keywords:** RNA-seq, fusion, cancer, FusionInspector, STAR-Fusion, Trinity

## Abstract

Here, we present FusionInspector for *in silico* characterization and interpretation of candidate fusion transcripts from RNA sequencing (RNA-seq) and exploration of their sequence and expression characteristics. We applied FusionInspector to thousands of tumor and normal transcriptomes and identified statistical and experimental features enriched among biologically impactful fusions. Through clustering and machine learning, we identified large collections of fusions potentially relevant to tumor and normal biological processes. We show that biologically relevant fusions are enriched for relatively high expression of the fusion transcript, imbalanced fusion allelic ratios, and canonical splicing patterns, and are deficient in sequence microhomologies between partner genes. We demonstrate that FusionInspector accurately validates fusion transcripts *in silico* and helps characterize numerous understudied fusions in tumor and normal tissue samples. FusionInspector is freely available as open source for screening, characterization, and visualization of candidate fusions via RNA-seq, and facilitates transparent explanation and interpretation of machine-learning predictions and their experimental sources.

## Highlights

• FusionInspector software for supervised analysis of candidate fusion transcripts
• Clustering of recurrent fusion transcripts resolves biologically relevant fusions
• Distinguishing characteristics of fusion transcripts in tumor and normal tissues

## Motivation

Gene fusions play a key role as driver oncogenes in tumors, and their reliable discovery and detection are important for cancer research, diagnostics, prognostics, and guiding personalized therapy. While discovering gene fusions from genome sequencing can be laborious and costly, the resulting fusion transcripts can be recovered from RNA-sequencing data of tumor and normal samples. However, alleged and putative fusion transcripts can also arise from multiple sources other than chromosomal rearrangements, including *cis*- or *trans-*splicing events, experimental artifacts during RNA sequencing, or computational errors of transcriptome reconstruction methods. Understanding how to discern, interpret, categorize, and verify predicted fusion transcripts is essential for consideration in clinical settings and prioritization for further research.

## Graphical abstract

**Figure:**
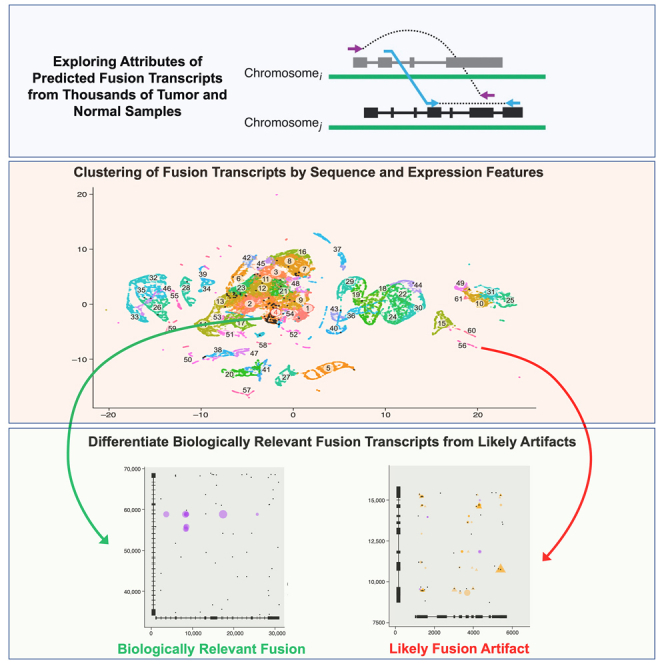

## Teaser

Haas et al. present FusionInspector, a method for *in silico* characterization and interpretation of candidate fusion transcripts identified via RNA sequencing. Applied to thousands of tumor and normal transcriptomes, FusionInspector identified numerous likely biologically relevant fusions with potential roles in cancer or normal cellular biology.

## Introduction

Gene fusions are intensely studied for their relevance to disease and normal cellular biology. In cancer, gene fusions typically result from chromosomal rearrangements, including well-known drivers of cancer, such as BCR::ABL1 in chronic myelogenous leukemia (CML),^1^^,^^2^ TMPRSS2::ERG in prostate cancer,^3^^,^^4^ and SS18::SSX1 or SS18::SSX2 in synovial sarcoma.^5^^,^^6^ Charting the diversity of fusion transcripts present in tumor and normal tissue is important for our basic understanding of the complexity and biological function of the transcriptome in normal and disease states, molecular diagnostics of cancer patients, and neoantigen discovery for targeting in personalized immunotherapy with cancer vaccines or T cell therapy.^7^^,^^8^

The structural rearrangements leading to gene fusions can be detected or inferred through whole-genome sequencing (WGS) or from the presence of “fusion transcripts” in whole-transcriptome RNA sequencing (RNA-seq).^9^^,^^10^ Given the easier and less costly nature of RNA-seq compared with WGS, and the effective methods for transcript assembly, RNA-seq has emerged as a leading experimental method for fusion transcript discovery and detection in both cancer research and molecular diagnostics. Dozens of computational tools have been developed to mine fusion transcripts from RNA-seq data (as referenced in Haas et al.^11^), and there have been multiple efforts to build catalogs of fusions across tumor and normal tissues.^12^^,^^13^^,^^14^^,^^15^^,^^16^^,^^17^ In general, tumor-specific fusion transcripts are presumed to derive from chromosomal rearrangements, whereas fusions identified in normal samples are more likely to be derived from normal karyotypes, thus reflecting other underlying causes, such as read-through transcription and *cis*- or *trans*-spliced products.

Nevertheless, predicting fusions from RNA-seq data is challenging, and the various methods developed to predict fusion products from RNA-seq vary tremendously in their accuracy for fusion detection, leading to both false positives and false negatives.^11^^,^^18^^,^^19^ False positives can be due to experimental artifacts that arise during reverse transcription or PCR amplification or computational mismapping of reads to target gene sequences,^20^ as well as specific differences in prediction tools. Moreover, as sequencing depth increases, the probability of detecting rare reads that support a fusion transcript prediction increases, due to either lab artifacts or real, low-rate *trans*-splicing of questionable functional relevance. Thus, there is an urgent need to understand the features that drive fusion detection and to generate high-quality catalogs of well-supported fusions.

**Figure 1:**
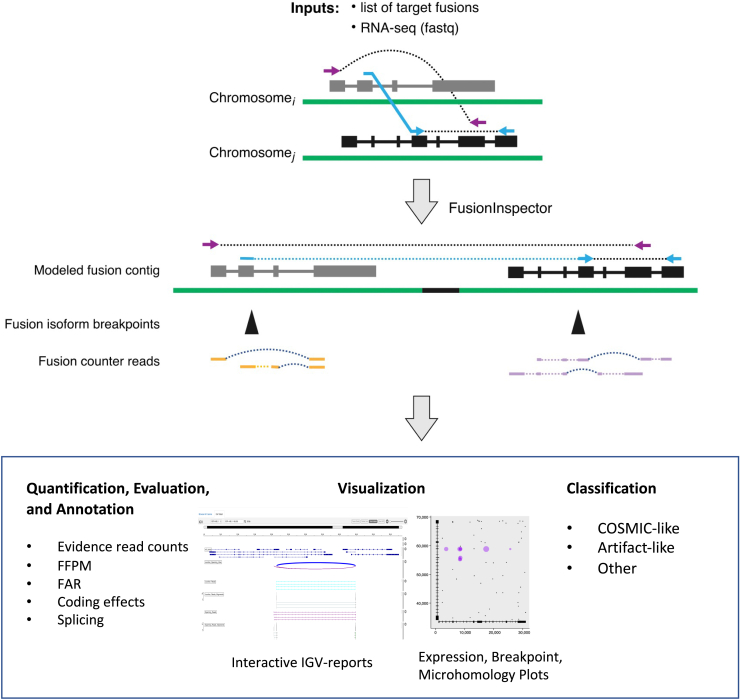
FusionInspector overview Top: lists of fusion candidates derived from predictions of one or multiple fusion detection methods, or from a screening panel, are provided to FusionInspector as input along with RNA-seq in fastq format. For each candidate fusion gene, fusion contigs are generated by fusing the full-length gene candidates as collinear on a single contig. Intronic regions are by default each shrunk to 1 kb. RNA-seq reads are then aligned to a reference consisting of the entire genome supplemented with fusion contigs. Fusion-derived reads that would normally align discordantly as chimeric alignments in the reference genome (top example) instead align concordantly in the fusion contig context (bottom example). Middle: FusionInspector identifies split-read alignments (light blue) and spanning pairs (purple) supporting the gene fusion in addition to read alignments that overlap the breakpoints and instead support the unfused fusion partners (fusion counter-reads). Bottom: for those fusions where FusionInspector captures RNA-seq read support (“*in silico* validation”), it reports fusion sequence and expression characteristics, interactive visualizations (Figure S1B), and predicted classifications as COSMIC-like, potential artifact, or other category (STAR Methods).

Here we describe FusionInspector (Figure 1), a method to assess and document the evidence for fusions. While other methods have previously been developed for fusion visualization,^21^^,^^22^^,^^23^^,^^24^^,^^25^ FusionInspector includes modules for supervised detection of fusion transcripts and comparing fusion transcripts with corresponding unfused fusion partners, and aims to differentiate likely biologically relevant fusions from likely experimental or bioinformatic artifacts. FusionInspector reassesses the read alignment evidence supporting targeted candidate fusion transcripts, comparing the relative alignment evidence for a fusion transcript with counter-evidence for its unfused partner transcripts. FusionInspector further evaluates the fusion transcript breakpoints in relation to sequence features representative of likely experimental and bioinformatic artifacts,^26^^,^^27^ including canonical splicing sequences, reference exon gene structures, and regions of microhomology between partner genes. Through reports, interactive visualizations, and classification, FusionInspector aims to *in silico* assess fusions for their likely validity and assist researchers in reasoning about the quantity and quality of the evidence supporting predicted fusions, to differentiate likely artifacts from fusions with characteristics similar to biologically relevant fusions known to occur in tumor and normal tissues. While potential oncogenicity and structural rearrangements may often be implied by tumor vs. normal sample prevalence and chromosomal loci for fusion gene partners, respectively, FusionInspector makes no assertions regarding oncogenicity or mechanism (genome rearrangement vs. *cis*- or *trans*-splicing) by which biologically relevant fusions may be derived.

We applied FusionInspector to assess recurrently predicted fusions in tumor and normal tissues, gathering insights into fusion transcript diversity and devising machine-learning methods for fusion classification (Figure S1A). We first applied FusionInspector to examine recurrently predicted fusions in tumor and normal tissues to compute sequence and expression features, from which we next generated clusters of fusion variants with similar characteristics. We then identified fusion clusters that are enriched for known biologically relevant fusions, and others as likely artifacts. From these fusion clusters, we trained a classifier to automatically predict fusion instances as likely biologically relevant, likely artifactual, or other type. Finally, we applied FusionInspector on additional predicted instances of fusion transcripts that were clustered with biologically relevant fusions and explored, classified, and prioritized these transcripts to identify additional relevant candidates in tumor and normal transcriptomes. Our application discovered a cluster of fusion transcripts heavily enriched for known cancer fusions and other fusions of interest. FusionInspector is freely available as open-source software at https://github.com/FusionInspector/FusionInspector/wiki.

## Results

### Development of FusionInspector for *in silico* evaluation of predicted fusion transcripts

FusionInspector (Figure 1) performs a supervised *in silico* evaluation of a specified set of candidate fusions, predicted from either RNA-seq data or from a user-defined panel. FusionInspector captures all read alignments in the RNA-seq that provide evidence for the specified fusions or for the unfused partner genes, and further explores the candidate fusion genes for regions of microhomology (defined as short identical sequence matches of length *k* [here *k* = 10]), and the proximity of microhomologies to putative fusion breakpoints.

To capture evidence supporting candidate fusions, FusionInspector identifies those reads that align concordantly between fusion genes as juxtaposed in their fused orientation and provide concordant alignments that span the two genes in this rearranged context. There are two types of fusion-supporting alignments: (1) split reads that define the fusion breakpoint, and (2) spanning fragments, where each paired-end read aligns to an opposite partner gene and the fragment bridges the fusion breakpoint (Figure 1). FusionInspector leverages the STAR aligner,^28^ which we enhanced here to support FusionInspector’s mode of action. As input to STAR, we provide the entire reference genome along with a set of fusion contigs constructed by FusionInspector (based on the list of specified fusion candidates), and STAR aligns reads to the combined genome targets and reports those aligned to the fusion contigs for further evaluation by FusionInspector (STAR Methods).

Next, FusionInspector computes several features that are associated with the fusion based on these alignments to assist in their evaluation. First, it uses the normalized number of reads exclusively supporting each fusion variant as a proxy for the expression of the fusion transcript (similarly, read alignments overlapping the fusion breakpoint and exclusively supporting the unfused partner genes are a proxy for the expression levels of the unfused partner genes). Second, it computes the fusion allelic ratio (FAR) for the fusion with respect to each (5′ or 3′) partner transcript (5′-FAR and 3′-FAR) as the ratio of mutually exclusive reads supporting the fusion variant vs. each unfused partner gene (Figures 1 and S1C). Third, it examines fusion breakpoints inferred from the read alignments for canonical dinucleotide splice sites at boundaries of the breakpoints in each partner gene and for agreement with available reference gene structure annotations. When there is evidence that supports multiple fusion transcript variants for a given fusion gene occurrence, FusionInspector uses an expectation-maximization-based algorithm to fractionally assign mutually compatible spanning fragments to the corresponding variants (STAR Methods). It then filters fusion variant candidates according to minimum evidence requirements (default settings require at least one split read to define the junction breakpoint, and at least 25 aligned bases supported by at least one read on both sides of the fusion breakpoint; STAR Methods). Finally, it captures microhomologies between putative fusion genes and determines the proximity of a fusion breakpoint to the nearest site of microhomology.

For clarity, we will use the following definitions when reporting on fusions cataloged among samples. “Fusion” refers to two genes, geneA and geneB, where there exists a fusion transcript involving exons from geneA and geneB, ordered respectively, and written as geneA::geneB according to the Human Genome Organization’s (HUGO) Gene Nomenclature Committee (HGNC) nomenclature for fusion genes.^29^ A “fusion occurrence” corresponds to a fusion identified in an individual sample, defined by the tuple (fusion, sample). A “fusion transcript” or “fusion variant” (used interchangeably) represents a fusion occurrence with a unique breakpoint (e.g., splicing isoform), defined by the unique tuple (fusion, sample, breakpoint coordinates). The primary fusion variant is defined as the variant with the highest expression level within an occurrence. Fusion occurrences and primary fusion variants are considered equivalent, and these terms are used interchangeably.

### Assessment of fusions using FusionInspector

**Figure 2:**
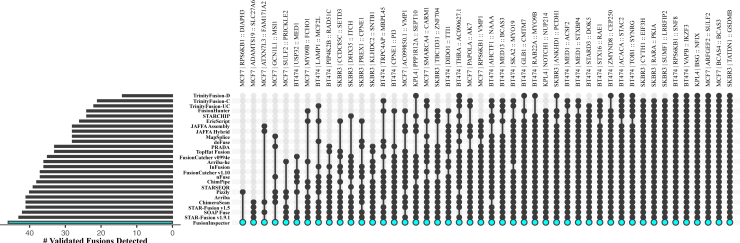
Detection of experimentally validated fusions in breast cancer cell lines BT474, MCF7, KPL4, and SKBR3 FusionInspector detects each of 46 experimentally validated fusions previously shown to be predicted by any of 24 different methods.^11^ FusionInspector results are highlighted as shown. While each sample was subject to inspecting identical lists of fusion candidates (STAR Methods), fusions were specifically identified only in the cell lines for which they are known to exist. See also Table S1.

By evaluating alignments to the modeled fusion contigs, FusionInspector demonstrates high sensitivity and specificity in supervised fusion detection, as we show for validated fusions in four well-studied breast cancer cell lines: BT474, KPL4, MCF7, and SKBR3. In our earlier study, 46 experimentally validated fusions were correctly predicted by at least one of 24 different fusion prediction methods.^11^ When FusionInspector was applied to the same data, it correctly identified each of these fusions in the relevant sample (Figure 2 and Table S1), with no false positives.

Using sequence and expression attributes of fusions and known characteristics of biologically relevant fusions (see below), FusionInspector further predicts whether each *in silico* validated fusion variant candidate (i.e., a fusion candidate evaluated by FusionInspector as having fusion read support evidence) is likely to be biologically relevant or alternatively has features consistent with experimental or bioinformatic fusion artifacts.

**Figure 3:**
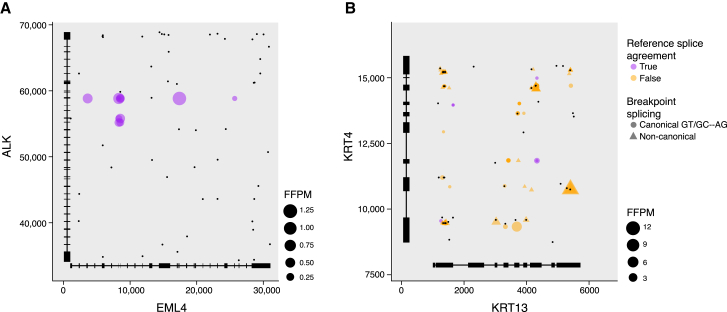
Features of fusion genes distinguish reliable and likely artifactual fusions Fusion variant expression level (dot size), splice type (dot color), and splice junction dinucleotide (dot shape) at each fusion breakpoint position involving the 5′ (x axis) and 3′ (y axis) partners of (A) EML::ALK (in COSMIC) and (B) KRT13::KRT4 (likely artifactual) fusions. Black dots: positions of microhomology (10 base exact match). Structures of collapsed splicing isoforms for fusion partner genes are drawn along each axis.

We illustrate these features in the context of two contrasting examples of fusion types (Figure 3). Fusion EML4:ALK, a known cancer driver prevalent in lung adenocarcinoma,^30^^,^^31^ has evidence of multiple transcript variant structures, and while microhomologies are found between the EML4 and ALK genes they tend to be distal from the fusion variant breakpoints (Figure 3A). The EML4::ALK fusion variant breakpoints are all found at consensus dinucleotide splice sites that coincide with exon boundaries of reference gene structure annotations. In contrast, FusionInspector captures many reads supporting a putative fusion KRT13::KRT4, but the breakpoints inferred from split-read alignments mostly have non-consensus dinucleotide splice sites and coincide with sites of microhomology; additionally, split reads with consensus dinucleotide splice sites mostly do not coincide with reference exon boundaries (Figure 3B). Because KRT13 and KRT4 are only distantly related with no easily detected nucleotide-level sequence conservation, their fusion may not be discarded by many fusion transcript predictors. However, given that most fusion evidence coincided with sites of microhomology and the lack of consensus splicing at breakpoints, FusionInspector infers most putative KRT13::KRT4 fusion variants to be artifactual. Another particularly compelling example of a similarly misleading and likely artifactual fusion is COL1A1::FN1, which is detected as prevalent in cancer-associated fibroblast cell lines (Figure S2). Further consideration of fusion and partner gene expression levels can aid in evaluating and prioritizing fusion candidates for further study, as we pursue below.

### Clustering of recurrent fusion transcripts via FusionInspector attributes resolves COSMIC-like and artifact-like fusions

**Figure 4:**
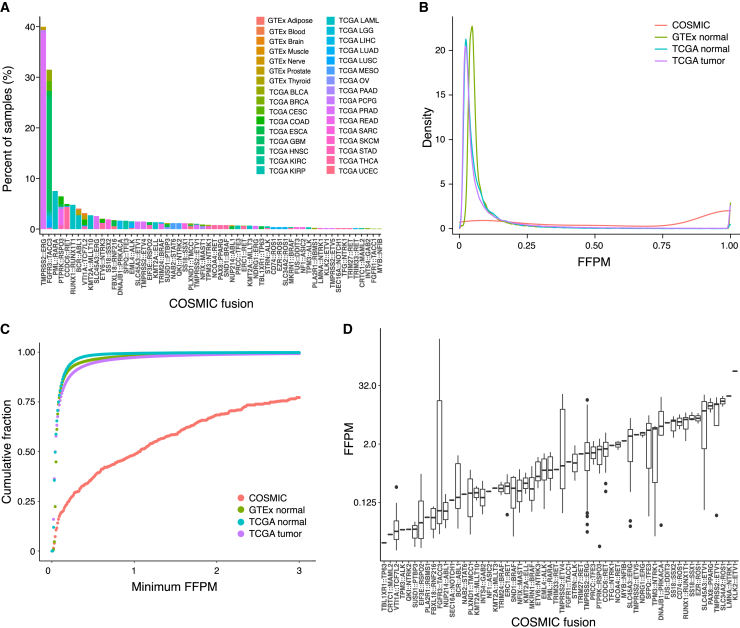
COSMIC fusions show distinctive properties among STAR-Fusion predictions across TCGA and GTEx (A) Tissue and tumor composition. Percentages of TCGA tumor or GTEx normal samples (y axis) with corresponding predicted COSMIC fusions (x axis). TCGA study abbreviation codes as in National Cancer Institute Genomic Data Commons.^38^ (B and C) COSMIC fusions are more highly expressed than other predicted fusions. (B–D) Expression levels. (B) Distribution of fusion expression levels (y axis, FFPM; right-truncated at 1 FFPM) for all fusions predicted in TCGA tumors (purple), TCGA normal (blue), GTEx (green), and COSMIC (red). (C) Cumulative fraction (y axis) of all predicted fusions at each minimum fusion expression (x axis, FFPM). (D) Distribution of fusion expression levels (y axis, FFPM) for each predicted COSMIC fusion (x axis). For (A)–(D), fusions are restricted to the single highest expressed fusion variant per sample occurrence, require reference annotation splice agreement at breakpoints, and have mitochondrial, HLA, and immunoglobulin-gene-containing fusions filtered. See also Table S2.

We first applied FusionInspector to examine recurrent fusion transcripts based on poly(A)-stranded RNA-seq from tumors (from The Cancer Genome Atlas [TCGA]^32^) and corresponding healthy tissue (from TCGA and Genotype-Tissue Expression [GTEx]^33^). To generate an initial comprehensive catalog of fusion variants in tumor and normal tissues, we predicted fusion transcripts with STAR-Fusion (v1.7) across 9,426 tumor and 707 normal samples from TCGA, and 8,375 normal samples from GTEx (Table S2). We initially applied lenient fusion evidence requirements to maximize sensitivity (STAR Methods). As a result, putative fusion transcripts were detected in nearly all tumor and normal samples. After applying a minimum expression level threshold (0.1 fusion fragments per million [FFPM]), we detected a significantly higher number of fusion occurrences in tumors vs. paired normal samples in several TCGA tumor types (Figure S3A), although there were similar median numbers of predicted fusion occurrences per sample type in TCGA tumor and GTEx normal samples (t test, p = 0.5, Figure S3B). We readily identified known cancer fusions included in the COSMIC fusion collection^34^^,^^35^ (“COSMIC fusions,” Figure 4A) according to known disease associations and prevalence, such as TMPRSS2::ERG identified in roughly half of prostate cancers,^3^ FGFR3::TACC3 in glioblastoma,^36^ and PML::RARA in the acute promyelocytic leukemia subtype of acute myeloid leukemia (AML).^37^ COSMIC fusions were more highly expressed than most predicted fusions, which had low estimated expression levels and few supporting reads (Figures 4B–4D).

Next, we used FusionInspector to examine the sequence and expression features of fusion transcripts that were recurrently detected across tumor and/or normal samples in order to distinguish biologically impactful fusions (akin to the COSMIC fusions) from experimental or computational artifacts, or from low levels of *cis*- or *trans-*splicing from highly expressed genes. To this end, we analyzed all 53,240 predicted fusion variants (38,591 fusion occurrences plus 14,649 alternative fusion variants) from 628 TCGA and 530 GTEx representative samples (STAR Methods). For each fusion candidate, FusionInspector identified the number of reads supporting the fusion variant and those supporting the unfused partner genes at putative breakpoints, identified regions of microhomology between partner genes, and determined the following features: inferred fusion expression level (FFPM), 5′ and 3′ fusion allelic ratios (5′-FAR, 3′-FAR), 5′ and 3′ unfused gene expression levels (5′-counter-FFPM and 3′-counter-FFPM), presence of consensus vs. non-consensus dinucleotide splice sites at fusion breakpoints, agreement or disagreement with reference gene structure exon boundaries at splice junctions, number of microhomologies observed between the two partner genes, and the distance of each inferred fusion breakpoint to the nearest site of microhomology.

**Figure 5:**
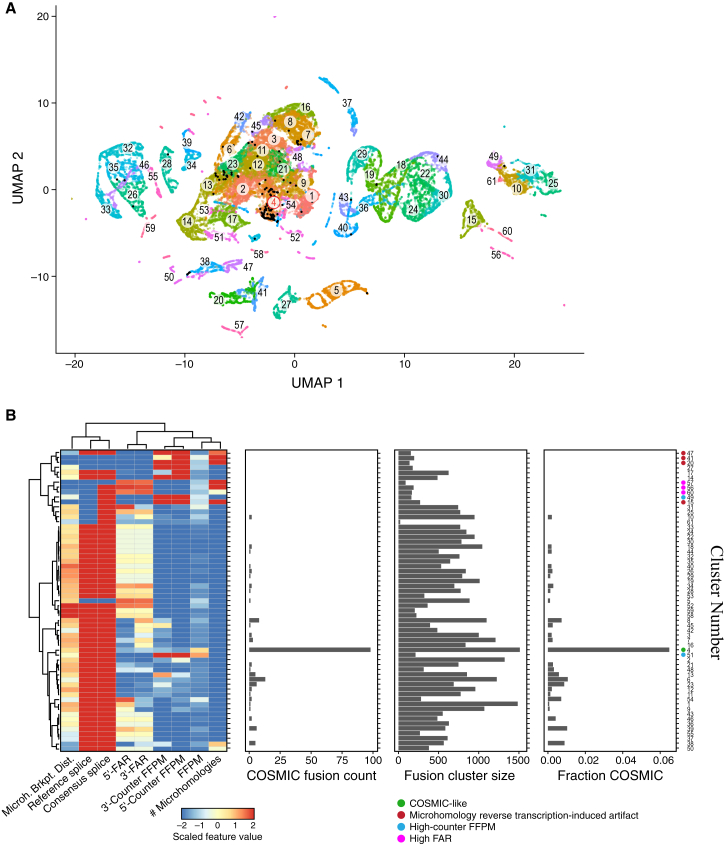
TCGA and GTEx fusion clustering by sequence and expression features distinguishes COSMIC-like fusions from likely artifactual ones (A) Fusion clusters. Uniform manifold approximation and projection (UMAP) of 53,240 TCGA and GTEx fusion variants feature profiles (dots), colored by Leiden cluster. Red label: cluster C4. (B) Cluster C4 is enriched for COSMIC fusions. Features (columns, right), number of COSMIC fusions (x axis, second from left), cluster size (x axis, second from right), and fraction of COSMIC fusions (x axis, right) for each fusion cluster (rows). Heatmap shows median scaled intensity values for each feature (color bar). See also Table S3.

To distinguish fusion artifacts from those with features consistent with biologically impactful fusions, we clustered fusion variants by their feature profiles (Figures 5A and S4; Table S3; STAR Methods). Clustering produced 61 high-granularity clusters, each containing an abundance of fusions identified in both TCGA and GTEx samples and distinguishable by feature attributes (Figures S5A–S5L). We further grouped these fusion variant clusters by hierarchical clustering according to median fusion attribute values in each fine cluster (Figure 5B). We then focused on examining clusters enriched for COSMIC fusions as a proxy for biologically impactful fusions, exploring those fusions with sequence and expression characteristics similar to those of COSMIC fusions, irrespective of potential genome rearrangement characteristics or known oncogenic roles.

One fusion cluster (C4) was significantly enriched with COSMIC fusions, harboring 57% of our detected instances of COSMIC fusion occurrences among these samples, but only 4% of all fusion occurrences (p < 10^−90^, Fisher’s exact one-sided test) (Figure 5B). Fusion variants in this cluster had splice breakpoints consistent with consensus splice sites and matching known reference gene structure exon boundaries, were relatively highly expressed, and were deficient in microhomologies between fusion partner genes. Most of the COSMIC fusions in C4 also have a 3′-FAR that exceeds the 5′-FAR, consistent with the fusion transcript being driven from an active 5′ partner’s promoter and a 3′ unfused partner expressed at lower levels (Figure S5M). Sixteen additional clusters, all but one (C10) of which are members of one large hierarchical cluster with related features, had at least two COSMIC fusion occurrences per cluster and spanned 34% of the fusion occurrences overall, including 37% of all COSMIC fusion occurrences.

Conversely, other fusion clusters, spanning 3% of all fusion occurrences and no COSMIC fusion occurrences, had features indicative of experimental or computational artifacts, especially enrichment in microhomologies that could confound alignment or contribute to reverse transcriptase mispriming. We thus consider those fusion variants as putative artifacts. Of the 3% of all fusion occurrences encompassed by these clusters, two-thirds (2% of all fusion occurrences) had moderately to highly expressed partner genes, suggesting origination from reverse transcriptase mispriming, and the remainder had little evidence for partner gene expression, suggesting read misalignment artifacts. The low portion of such presumed artifacts is a testament to STAR-Fusion’s rigorous filtering.^11^ A further 1% of fusions involved highly expressed partner genes, where the detected fusion represented a small fraction of the total expression from these loci. These fusions may result from low levels of *cis*- or *trans-*splicing from the highly expressed partner genes.

### A fusion classifier allows targeted screening of predicted COSMIC-like fusions

We reasoned that the set of 1,511 predicted fusion occurrences (835 distinct fusions) that were members of the COSMIC-enriched cluster C4 are likely enriched for fusions of functional significance and should be prioritized for further study. Some are already known to be relevant to cancer but not yet included in the COSMIC database, such as EGFR::SEPT14,^39^, PVT1::MYC,^40^^,^^41^^,^^42^ and TPM3::NTRK1.^43^ Others are reciprocal fusions for COSMIC fusions that could result from balanced translocations, including reciprocal ABL1::BCR1 of COSMIC BCR1::ABL1, BRAF::SND1 of COSMIC SND1::BRAF, and PPARG::PAX8 of COSMIC PAX8::PPARG. This fusion cluster is also enriched for fusions exclusively identified in pancreatic tissue (explored below).

**Figure 6:**
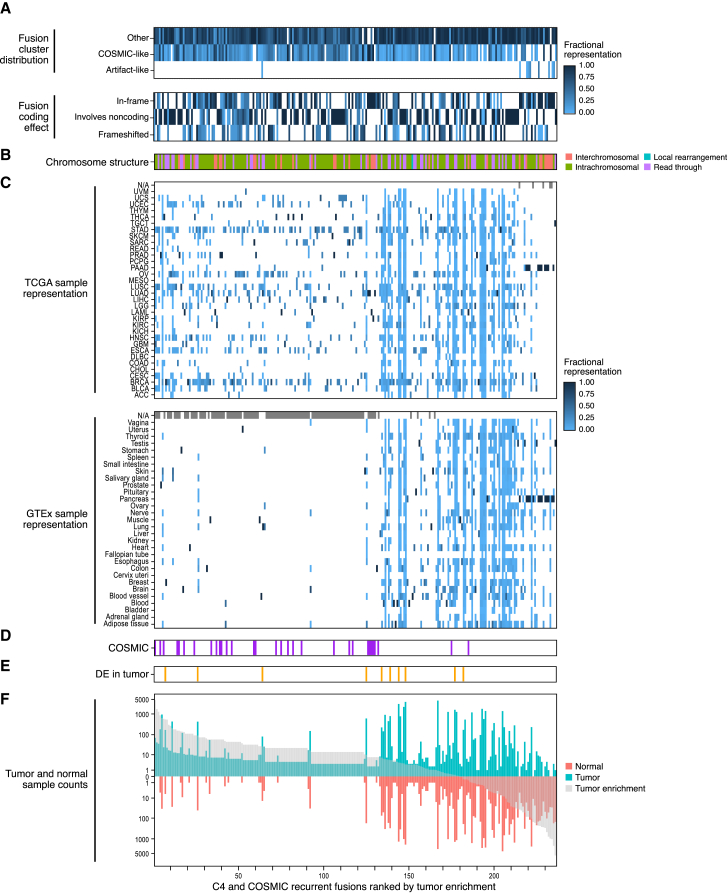
Characteristic properties of recurrent TCGA and GTEx COSMIC and COSMIC-like fusions can distinguish biologically meaningful fusions and fusion instances Two hundred thirty-six selected COSMIC-like (C4) and additional COSMIC fusions (columns/x axis) rank ordered by tumor enrichment and shown with fraction of the instances of each fusion in each category based on predicted Leiden cluster labels (A, rows, top) or corresponding to presumed impact on coding sequence (A, rows, bottom); fusion structure type based on the fusion partner’s chromosomal location (B); fraction of instances that is in each tumor or tissue type in TCGA and GTEx (C, rows); presence in COSMIC (D, purple); significantly higher expression in tumors vs. normal tissues (E, Wilcoxon rank-sum test applied to FFPM requiring a minimum of three tumor and three normal samples, Benjamini-Hochberg FDR < 0.05 and median tumor FFPM > median normal FFPM, orange); number of tumor (sea green) or normal (light red) samples (F, y axis) predicted by STAR-Fusion to contain the fusion, rank ordered by tumor enrichment (F, x axis, (STAR Methods, gray). Computations in (A), (B), and (D) are output by FusionInspector. See Figures S6A–S6E for each fusion pair. See also Table S4.

To gain further insights into the characteristics of the COSMIC-like fusions in C4, we first screened additional TCGA and GTEx samples to characterize additional occurrences of C4 representative fusions. We refocused FusionInspector on 236 C4+ key fusions (231 C4 recurrent fusion gene pairs including 26 recurrent COSMIC fusions, plus another five recurrent COSMIC fusions having all occurrences localized outside of C4; STAR Methods and Table S4^44^). We screened each of 2,764 TCGA and 1,009 GTEx representative samples for these 236 fusions (STAR Methods), collecting FusionInspector *in silico* validations and attributes for 37,211 additional fusion occurrences (Figures 6 and S6A–S6E; Table S4), and ranked fusions by the difference in their initial detected prevalence in tumors vs. normal samples.

To determine whether additional occurrences of these fusions have characteristics consistent with COSMIC-like C4 fusions, are artifact-like, or belong to another category, we trained a random forest classifier to predict the labels of each of the earlier-defined 61 Leiden clusters and applied it to predict the cluster labels of fusion variants examined in this expanded targeted survey. We then categorized each fusion occurrence by the overall category of the hierarchical cluster to which the Leiden cluster label it was classified to belongs (e.g., “COSMIC-like” for C4 predictions) (Figure 6A and STAR Methods). Evaluation of this random forest classifier using 5-fold cross-validation and application to biological replicates demonstrates high prediction accuracy, with most clusters yielding >90% prediction accuracy and mispredicted fusion variants or biological replicate fusion variants assigned to fusion clusters with highly similar features, effectively discriminating artifact-like fusions from others (Figure S7). This fusion classification was further incorporated into FusionInspector for routine application in discriminating COSMIC-like fusions from artifacts or other types.

FusionInspector-screened occurrences of known COSMIC fusions were mostly tumor enriched with few to no normal samples identified with evidence (noting that all 1,009 GTEx normal samples were screened by FusionInspector for an identical list of COSMIC fusions). All but 31 of the 236 fusions had occurrences classified as C4. Only seven fusions had at least 10% of their occurrences predicted to clusters with high counter-evidence (C49 or C51), suggesting the fusion transcripts may reflect low levels of *cis*- or *trans*-splicing of more highly expressed normal fusion partner genes. Only nine fusions had any occurrences predicted as artifacts, found in both TCGA and GTEx as pancreas-specific fusions (further discussed below).

This analysis highlighted intriguing, well-supported fusions for further study. For example, while the top-ranked tumor-enriched fusion, FGFR3::TACC3 (rank 1, 70 tumor samples, 0 normal) is a known oncogenic driver,^36^ other top-ranking fusions, such as CCAT1:CASC8 (rank 2, 42 tumors—mostly lung and stomach cancers, 0 normal) and VCL::ADK (rank 3, 36 tumors—also mostly lung and stomach cancers, 0 normal) have not yet been extensively studied. CCAT1::CASC8 was only recently reported in the fusion catalog generated by DEEPEST fusion,^17^ and VCL::ADK was only previously reported in a study of cancer cell lines.^12^

Because our initial selection of samples for FusionInspector exploration of recurrent fusion transcripts did not strictly require COSMIC fusions, only 31 of the 58 known COSMIC fusions initially discovered by our comprehensive survey with STAR-Fusion were further characterized above. We thus further applied FusionInspector to 46 additional samples with instances of the remaining 27 COSMIC fusions. Over 80% of these COSMIC fusion occurrences were predicted to other COSMIC fusion-containing clusters, none as likely artifacts, and 11 (41%) of these had occurrences predicted to the COSMIC-enriched cluster C4 (Figure S8A and Table S5), including KLK2::ETV1, LMNA::NTRK1, RUNX1::RUNX1T1, FUS::DDIT3, TMPRSS2::ETV5, MYB::NFIB, TFG::NTRK1, TRIM27::RET, MKRN1::BRAF, INTS4::GAB2, and NUP214::ABL1.

### Some fusion transcripts are prevalent in normal tissues and may not be oncogenic

Approximately one-third (86) of the 236 C4+ targeted fusions in our analysis were robustly detected in normal tissues (found in at least five normal samples); these may not be particularly relevant to cancer biology but may play a role in normal biological processes. Of these, 61 fusions are broadly expressed across at least five tissues, involve intrachromosomal pairs of genes, and can be largely explained by read-through transcription, local rearrangements, or *trans-*splicing of neighboring transcripts.

Some other putative fusions that are prevalent in normal tissues may in fact represent normal structural variation in the human genome, which is not accounted for when performing read alignment to a single human reference. For example, Fusion KANSL1::ARL17, which would require a local rearrangement in the human reference genome, is prevalent across both tumor and normal tissues (median of 31% of individuals, Figure S8B), and is known to correspond to a common haplotype involving a locally rearranged genomic region observed in populations of European descent.^45^ An earlier report identified KANSL1::ARL17 in diverse tumor samples and proposed that it may be a cancer predisposition germline fusion specific to Europeans.^46^ Note, however, that no specific human genetic association evidence was shown for predisposition thus far, and we observe slightly higher prevalence of KANSL::ARL17 among GTEx normal samples than in tumors from TCGA (Figure S8B). Another normal fusion due to a rarer germline structural variation is TFG::GPR128, previously associated with a copy number variation and a haplotype frequency estimated at around 2% of individuals of European descent.^47^ Consistently, we find TFG::GPR128 broadly expressed across tumor and normal tissues and represented similarly at a median of 2% of all tissues examined (Figure S8C). As more evidence of common structural variation becomes available, other prevalent fusions found in normal tissues may be more easily explained.

Another set of fusions that are less easily explained involve those we found only in normal pancreas and pancreatic carcinoma (Figures 6 and S6), involving various pairwise combination of CPA1, CPA2, CLPS, CELA2A, CELA3A, CTRB1, CTRB2, and CTRC (e.g., CELA3A::CPA2, CELA3B::CELA2A, and CELA3A::CELA2A) fused to generate in-frame fusion products. These genes are among the highest expressed in pancreas and mostly reside on different chromosomes, suggesting that *trans*-splicing may be the predominant underlying mechanism. While the initially detected transcripts were within the COSMIC-peak-enriched fusion cluster, the random-forest-based fusion classifier did not predict the additional instances as COSMIC-like, and some of them (e.g., CELA3A::CTRC) have high fractions of occurrences predicted to “high-counter-evidence” clusters or “artifact-like” types (Figures 6 and S6E; Table S4).

### Some well-established oncogenic fusions are also reliably detected in normal samples

Several of the COSMIC fusions or other tumor-enriched fusions with known ties to cancer were surprisingly identified in both tumor and normal samples. For example, the prostate cancer fusion TMPRSS2::ERG, identified as our fourth most tumor-enriched fusion (182 of 465 TCGA prostate tumor samples), is also detected in six normal prostate samples (five TCGA and one GTEx) (Figure S6A). Each of the five TCGA prostate normal samples containing TMPRSS2::ERG were found with TMPRSS2::ERG in their matching prostate tumor samples, likely due to tumor-in-normal contamination. TMPRSS2::ERG was only identified by FusionInspector in prostate tumors or normal prostate, reflecting both this fusion’s high tissue specificity and FusionInspector’s high specificity of fusion calling.

In another example, COSMIC fusion PVT1:MYC was originally identified by STAR-Fusion in 20 samples (14 TCGA tumor, 1 TCGA normal, and 5 GTEx samples). Interactions between PVT1 and MYC including their fusion are well-known contributors to tumorigenesis.^40^^,^^41^^,^^42^ Through subsequent screening of PVT1::MYC with FusionInspector, we identify a total of 32 samples (+9 TCGA tumor, +1 TCGA normal, and +3, −1 GTEx). Most (21/32) are expressed at low levels (below 0.1 FFPM), and we do not find strong evidence for expression to be generally higher in tumor samples than in normals (p < 0.07, Wilcoxon rank-sum test). However, 5 of the 32 PVT1::MYC occurrences were identified in cervical cancer tumors, and all were significantly more highly expressed than the other samples (p < 0.001), with the most highly expressed at 19 FFPM (Figure S8D). PVT1 and MYC are co-localized to a proximal region in the bottom arm of chromosome 8, and a PVT1::MYC fusion would likely involve local restructuring at the locus in tumors to generate the fusion product. Interestingly, this chromosome 8 region is a known hotspot for insertion of human papilloma virus (HPV),^48^ the leading cause of cervical cancer (>90% of cases). Most of the TCGA cervical cancer samples we identified with PVT1::MYC have HPV insertions at this hotspot (see Table S3 of the study by TCGA Research Network^48^). Thus, we hypothesize that HPV insertion contributes to the formation of the PVT1::MYC fusions. We did not find evidence for HPV insertion in the breast cancer sample with similar levels of PVT1::MYC expression (data not shown).

COSMIC fusion VTI1A::TCF7L2, originally identified as an oncogenic fusion in colorectal cancer,^49^ was most abundant in stomach, colon, and esophageal carcinoma samples but was also detected in seven individual GTEx normal samples (brain, whole blood, tibial nerve, tibial artery, prostate, and breast) (Table S4). While VTI1A::TCF7L2 was not enriched for detection in tumors vs. normal, only those fusions in colon cancer were highly expressed (>0.15 FFPM), whereas other tumor and normal instances were lowly expressed (<0.05 FFPM; many at the limit of detection; Figure S8E), supported by a single split read defining the fusion breakpoint (Table S4). This could be consistent with a very low proportion of cells in the normal tissue expressing the fusion, compared with a large clone in the tumor.

Interestingly, COSMIC fusion BCR::ABL1 was not tumor enriched in our analysis, likely due to paucity of the relevant tumors in TCGA. In particular, BCR::ABL1 occurs in >95% of CML cases,^1^^,^^2^ but TCGA lacks CML samples. Indeed, the four TCGA tumors with BCR::ABL1 likely correspond to a subtype of AML defined with this fusion.^50^ Three of these AML tumors also have evidence of the reciprocal ABL1::BCR fusion, and the oncogenic BCR::ABL1 is expressed at higher levels than the reciprocal counterpart in each sample. Interestingly, we detected eight instances of the oncogenic BCR::ABL1 fusion (and none of the reciprocal) in six different GTEx normal tissues (one each of adipose, breast, nerve, prostate, and thyroid, and two pancreas) and one TCGA normal kidney sample. We observed no sequence or expression features distinguishing these fusions from those we identified in AML, and fusion transcript breakpoints for those in GTEx normal tissues are identical to those found in AML. In general, when we find COSMIC fusions in GTEx normal samples, they are found at low frequencies (<1% prevalence in a tissue type).

### FusionInspector analysis of fusion transcripts in pediatric cancers

To highlight the utility of FusionInspector, we applied it to the 1,366 RNA-seq datasets from 1,230 participants across seven pediatric cancer projects in the Therapeutically Applicable Research to Generate Effective Treatments (TARGET) resource.^51^ These include acute lymphoblastic leukemia (ALL; 666 tumors), AML (217 tumors and 20 normal samples), clear cell sarcoma of the kidney (CCSK; 13 tumors), neuroblastoma (NB; 157 tumors), osteosarcoma (OS; 88 tumors), rhabdoid tumor (RT, 63 tumors and 6 normal), and Wilms’ tumor (WT; 130 tumors and 6 normal).

STAR-Fusion identified 213,786 fusion occurrences involving 108,661 distinct fusion gene pairs (Table S6) with 3 (RT) to 19 (CCSK) median fusions detected (minimum 0.1 FFPM) per tumor sample, and 2.5–3.5 median fusions per normal sample (similar to normal samples in TCGA or GTEx). Eighty-six percent of the STAR-Fusion predicted occurrences were *in silico* supported by FusionInspector, yielding 182,807 fusion occurrences with 217,614 fusion variants involving 88,307 distinct fusion gene pairs (Table S6), including 30 COSMIC fusion gene pairs. Some of the most prevalent included known pediatric cancer fusions RUNX1::RUNX1T1 (15% AML participants), KMT2A::MLLT3/MLLT10 (12% AML), TCF3::PBX1 (4.4% ALL), and ETV6::RUNX1 (2.4% ALL). Of these 30 TARGET COSMIC fusions, 22 were only found among the TARGET pediatric tumors with the remaining eight also found among TCGA samples (Figure S9A–S9C).

**Figure 7:**
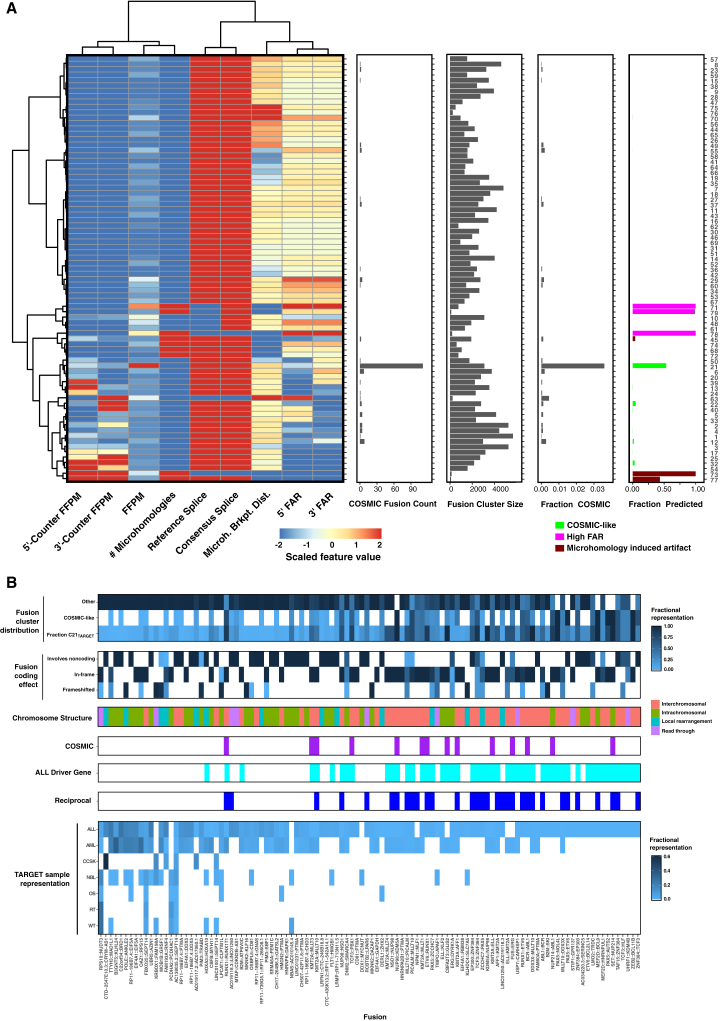
TARGET pediatric cancer fusion clusters and attributes (A) TARGET pediatric fusion clustering by sequence and expression features yields a COSMIC-enriched fusion cluster. Leiden clustering of 217,614 fusion variants yielded 79 clusters. Cluster 21 was enriched for known COSMIC fusion occurrences. From left to right: median scaled attribute features for clustered fusions, counts of COSMIC fusion occurrences per cluster, counts of clustered fusion occurrences, fraction of fusion occurrences corresponding to COSMIC fusions, and fraction of occurrences with predicted fusion cluster attribute based on the earlier-trained random forest predictor. (B) Recurrent TARGET COSMIC and COSMIC-like fusions include additional fusions potentially relevant to pediatric cancer. Fusion characteristics are shown for 108 selected recurrent fusions from cluster C21_TARGET_. Fusion cluster distribution, fractions of predicted COSMIC-like or OTHER and observed fractional C21_TARGET_ fusion occurrence representation; Fusion coding effect, impact of fusion on the coding region leading to in-frame coding regions, frameshifting, or involving noncoding regions at fusion occurrence breakpoints; Chromosome structure, fusion structure type based on the fusion partner’s chromosomal location. COSMIC (purple) indicates presence in COSMIC; ALL Driver Gene (cyan) indicates whether either fusion gene partner is an earlier-defined ALL cancer driver gene (as per Brady et al.^52^); reciprocal (dark blue) indicates whether the fusion gene is found in reciprocal form within an individual participant. TARGET sample representation: fraction of pediatric cancer tumors with corresponding fusion occurrences. Fusions are shown ranked from left to right according to cumulative fraction of pediatric cancer type prevalence. See also Table S6.

The random forest classifier classified 1.6% of the 217,614 fusion variants as COSMIC-like and 1.2% as artifact-like. The fusion variants partitioned into 79 clusters, one of which (C21_TARGET_), containing 3,091 occurrences of 910 fusions, was significantly enriched for COSMIC fusions (p < 10^−150^, one-sided Fisher’s exact test) (Figures 7A and S9D), including 22 of the 30 COSMIC fusions. These included hallmark fusions RUNX1::RUNX1T1, TCF3::PBX1, ETV6::RUNX1, and several KTM2A-partnered fusions. Some samples had evidence of reciprocal fusion partners, including BCR::ABL1 and ABL1::BCR (Figure 7B). The fusions in C21_TARGET_ had sequence and expression features consistent with the COSMIC-enriched cluster C4 of TCGA and GTEx fusions, especially high fusion expression, and 3′-FAR generally exceeding 5′-FAR (Figure 7A).

We examined C21_TARGET_ fusions to identify other fusions potentially relevant to pediatric cancer. Of 910 fusions, 108 recurrent fusion candidates were present in at least three samples and absent from normal samples (Figure 7B and Table S6). These include fusions not yet reported in the COSMIC oncogenic fusion database but otherwise known as drivers or highly relevant to pediatric oncology,^51^^,^^52^ such as TCF3::HLF,^53^^,^^54^^,^^55^ ZNF384::EP300,^56^ and CBFB::MYH11.^57^ Many of the 108 fusions (51 fusions = 47%) involve a known ALL driver as one or both partners,^52^ such as KMT2A (eight fusions); PAX5, ZNF384, and MLLT10 (five fusions each); and NUP98, TCF3, and ETV6 (four fusions each). Most driver-gene-containing fusions (28/51 = 55%) involve reciprocal fusion arrangements. Other fusion partners frequently found among this set that may be potentially relevant to pediatric cancer include PTMA (seven fusions), EIF4A1 and the EIF4A1 locus-derived RP11-186B7.4 (seven unique fusions), and SEPT14 (three fusions).

While our version of FusionInspector was not compatible with exploration of immunoglobulin heavy chain gene (IGH) fusions given the large IGH locus size (1.3 Mb vs. our 100 kb maximum intron length; STAR Methods), 12 (1.8%) TARGET ALL samples had evidence of IGH::CRLF2 fusions, a common oncogenic IGH fusion.^58^ We also found the oncogenic ALL fusion P2RY8::CRLF2^59^ in 25 (3.8%) ALL participants. These were members of C10_TARGET_, likely due to consensus splice breakpoints that did not coincide with reference gene structure exon boundaries, highlighting the relevance of other clusters as sources of biologically relevant fusions with alternative non-artifactual attribute profiles.

## Discussion

We developed FusionInspector to enable exploration of the evidence supporting candidate fusions, flag likely artifacts, and identify those fusions with sequence and expression features similar to known biologically relevant fusion transcripts irrespective of oncogenicity or potential genome structural rearrangements. Given a list of candidate fusions, FusionInspector captures RNA-seq read alignments that support either the fused genes or the unfused partner genes. From the fusion and partner gene expression evidence coupled with sequence features relating to the fusion breakpoint, FusionInspector helps the user to reason about the nature and quality of any target fusion transcript.

Clustering TCGA and GTEx fusions by shared sequence and expression features identified a cluster of fusions highly enriched for COSMIC fusions. Fusions in the COSMIC-enriched cluster had relatively high fusion expression with 3′-FAR generally exceeding 5′-FAR, suggesting oncogenic activity from the 3′-fused transcript. Analysis initiated by fusions in the COSMIC-enriched cluster highlighted several putative or less appreciated oncogenic fusions, including CCAT1::CASC8 and VCL::ADK, based on their feature similarity to other well-known tumor-enriched fusions. Only ∼3% of initially predicted fusions were members of clusters likely enriched for artifacts based on features such as high partner gene expression or sites of microhomology at or near the fusion breakpoint. The low artifact rate is likely due to the strong filtering of the initial input catalog from STAR-Fusion.

We obtained similar results when applying FusionInspector to the TARGET pediatric cancer cohorts, with a single COSMIC-enriched cluster of fusion occurrences with relatively high fusion expression and 3′-FAR, containing known cancer drivers and yielding a source of additional potential fusion genes for future study. These additional fusions included gene partners PTMA, EIF4A1, and SEPT14, each with known roles in cancer (e.g., Frattini et al.,^39^^,^^60^^,^^61^ Kumar et al.,^39^^,^^60^^,^^61^ and Wu et al.^39^^,^^60^^,^^61^) but yet to be further characterized as pediatric cancer fusion transcripts.

While we focused on fusions identified in COSMIC-enriched clusters, other fusion clusters also harbor important oncogenic fusions. For example, the COSMIC fusions SS18::SSX1 and SS18::SSX2, known drivers of synovial sarcoma,^5^^,^^62^ are in other clusters (C38 and C39), due in part to their higher 5′-FAR. Another fusion of interest, FSIP1::RP11-624L4.1, is present in 240 (22%) of breast tumors analyzed and in 16 normal breast tissues samples, where it is expressed at significantly lower levels in normal tissues (Figure S10). While the individual fusion partners have cancer associations,^63^^,^^64^ any role for this likely read-through/*cis*-spliced fusion transcript deserves consideration in further exploring the roles of both genes in disease.

In some cases, fusions that were found in both tumor and normal tissues might reflect a low level of oncogenic events. For example, hallmark driver fusions including TMPRSS2::ERG and BCR::ABL1 were also detected in GTEx normal tissue samples, which may reflect a low proportion of premalignant or transformed cells.^65^^,^^66^^,^^67^ We also detected COSMIC fusion VTI1A::TCF7L2 across multiple tumor and normal tissue types (consistent with Nome et al.^68^), but only highly expressed in colon cancer samples where it is a postulated oncogenic driver.^49^^,^^69^ Whether such a fusion could contribute to tumorigenesis in a different tissue with different cellular circuitry remains unknown. While we are intrigued by evidence of oncogenic fusions in normal tissue RNA-seq data, we have not ruled out explanations such as tumor-in-normal contamination^70^ or potential cross-sample sequencing read contamination.^71^

Fusions that were prevalent among normal tissues can mostly be explained by read-through transcription and *cis*-splicing of co-linear genes, but some may simply reflect natural germline structural variations that may exist in the population. With ongoing advancements in methods for detecting and cataloging of structural variants,^72^^,^^73^ we may soon better understand the structural basis for many naturally occurring fusion transcripts. Access to matched RNA-seq and WGS of the same samples across individuals would greatly facilitate such efforts.

Pancreas stood out as a clear outlier among all normal tissues explored for fusions. While we suspect some of the putative fusions detected in pancreas are derived from reverse transcription or alignment artifacts, several did have features consistent with *trans-*splicing of highly expressed partner genes, with *trans*-spliced products yielding in-frame proteins. In general, these fusion occurrences do not have COSMIC-like sequence and expression features. *Trans*-spliced in-frame fusion transcripts have the potential to expand functional diversity from our otherwise linear genomes,^74^ and even if these pancreas-specific candidates failed to ultimately reach our COSMIC-like prioritization status, they may be worth additional studies.

FusionInspector opens the way to further explore the biological impact of the predicted fusions and the tissues and gene expression networks in which they are phenotypically relevant. FusionInspector helps illuminate the evidence supporting fusions in RNA-seq or to screen for relevant fusions sensitively and accurately in samples of interest. While expression attribute analysis and fusion class prediction by FusionInspector should require poly(A)-stranded RNA-seq, the supervised fusion variant identification could leverage alternative RNA-seq methodologies.

Because short reads remain limited in their capacity to represent full-length fusion transcripts, FusionInspector further integrates Trinity^75^^,^^76^ for *de novo* reconstruction to optionally reconstruct more full-length fusion transcripts from RNA-seq data aligned to each fusion contig. FusionInspector is available as a stand-alone application for screening lists of candidate fusion transcripts and is also incorporated into STAR-Fusion for *in silico* validation or visualization of STAR-Fusion-predicted fusion transcripts. This facilitates analysis of fusions from both bulk and single-cell RNA-seq, as we have recently demonstrated.^77^

Long-read transcriptome sequencing may eventually obviate short-read sequencing for fusion detection, thus removing the need for *de novo* reconstruction of full-length fusion transcripts.^78^^,^^79^ Full-length single-molecule direct RNA-seq^80^ should also avoid reverse transcription amplification artifacts. Conversely, other features scored by FusionInspector, such as expression characteristics of fusion transcripts with respect to partner genes, will remain relevant and easily adapted for long-read RNA-seq.

Machine learning is likely to play an increasingly important role in biomedical science and its clinical applications. In this paper we emphasize an important companion direction to machine learning, namely generating transparent and interpretable predictions, loosely referred to as explanations. The area of explanations and causal interpretation is growing rapidly in artificial intelligence.^81^ We need to keep a reproducible trace of facts, predictions, and hypotheses from gene to function in the era of big data.

We hope that the practical applicability of FusionInspector will help drive transparency and other explanatory efforts in predictive areas in genomics and personalized medicine more generally, including screening and reassessment of evidence supporting fusion predictions, and visualization of the evidence via interactive reports (Figures 1 and S1B). FusionInspector can be easily leveraged as an add-on component to any fusion transcript prediction pipeline, and is directly incorporated into STAR-Fusion to facilitate execution as part of the Trinity Cancer Transcriptome Analysis toolkit.^82^ The FusionInspector software is freely available as open source on GitHub (key resources table), provided in container form via Docker and Singularity, and accessible on the Terra cloud computing framework for secure and scalable application across large compendia of sample collections or patient-derived RNA-seq data.

### Limitations of the study

FusionInspector as implemented here requires read alignment overlap with exons of reference gene structure annotations, and hence fusions restricted to intronic regions of either fusion partner or neighboring intergenic regions presently go undetected. Because genome-level fusion events often occur outside of exons, FusionInspector is largely ineffective with whole-genome or whole-exome sequencing data, instead necessitating RNA-seq.

## STAR★Methods

### Key resources table

**Table:** 

REAGENT or RESOURCE	SOURCE	IDENTIFIER
**Deposited data**		

TCGA RNA-seq	http://Terra.bio	dbGaP: phs000854
Breast cancer cell lines RNA-seq for BT474, KPL4, MCF7, and SKBR3	SRA	SRA:SRP003186
GTEx RNA-seq	http://Terra.bio	dbGaP: phs000424
TARGET RNA-seq	http://Terra.bio	dbGaP: Acute Lymphoblastic Leukemia: phs000463, phs000464; Acute Myeloid Leukemia (AML): phs000465; Clear Cell Sarcoma of the Kidney (CCSK): phs000466; Neuroblastoma (NB): phs000467; Osteosarcoma (OS): phs000468; Rhabdoid Tumor (RT): phs000470; Wilms Tumor (WT): phs000471

**Software and algorithms**		

FusionInspector v2.4.0	This paper	https://github.com/FusionInspector/FusionInspector/releases/tag/FusionInspector-v2.4.0 or https://doi.org/10.5281/zenodo.7791682
STAR-Fusion v1.7	GitHub	https://github.com/STAR-Fusion/STAR-Fusion/releases/tag/v1.7.0
STAR-Fusion v1.9.1	GitHub	https://github.com/STAR-Fusion/STAR-Fusion/releases/tag/v1.9.1

**Other**		

Supplementary code	This paper	https://github.com/broadinstitute/FusionInspectorPaper or https://doi.org/10.5281/zenodo.7791682

### Resource availability

#### Lead contact

Further information and requests for resources or code should be directed to lead contact Brian Haas (bhaas@broadinstitute.org).

#### Materials availability

This study did not generate new unique reagents.

### Method details

#### Initial comprehensive fusion transcript survey for TCGA and GTEx via STAR-Fusion

Fusions were predicted for TCGA and GTEx samples using STAR-Fusion (v1.7). First, the STAR (v2.6.1a) aligner was used to align RNA-seq reads from each sample to the human genome as follows:

"STAR --genomeDir ctat_genome_lib_build_dir/ref_genome.fa.star.idx --outReadsUnmapped None --chimSegmentMin 12 --chimJunctionOverhangMin 12 --chimOutJunctionFormat 1 --alignSJDBoverhangMin 10 --alignMatesGapMax 100000 --alignIntronMax 100000 --alignSJstitchMismatchNmax 5 -1 5 5 --runThreadN 16 --outSAMstrandField intronMotif --outSAMunmapped Within --outSAMtype BAM Unsorted --readFilesIn reads_1.fastq reqds_2.fastq --outSAMattrRGline ID:GRPundef --chimMultimapScoreRange 10 --chimMultimapNmax 10 --chimNonchimScoreDropMin 10 --peOverlapNbasesMin 12 --peOverlapMMp 0.1 --genomeLoad NoSharedMemory --twopassMode Basic.

The resulting Chimeric.out.junction files generated by STAR containing candidate chimeric reads were then analyzed by STAR-Fusion like so "STAR-Fusion -J Chimeric.out.junction -O $output_dir/STARF --genome_lib_dir $ctat_genome_lib --min_FFPM 0 --no_annotation_filter" leveraging CTAT genome library GRCh38_gencode_v22_CTAT_lib_Sept032019.

The parameters used here eliminated any filtering of fusions according to fusion expression levels or based on fusion annotations, to retain any fusions known to frequently occur in normal samples in both the normal and the tumor samples for further study. All STAR-Fusion predictions are provided in Table S2.

Fusion tumor enrichment was computed for each fusion according to ( (# tumor with fusion + 1) / (total tumor samples)) / ( (# normal with fusion + 1) / (total normal samples) ).

#### FusionInspector method and implementation

FusionInspector takes as input a list of candidate fusions and RNA-seq files in fastq format, with each fusion formatted as "geneA::geneB" indicating a candidate fusion between geneA (5’) and geneB (3). Leveraging the companion CTAT genome library set of genomic resources (identical to that used with STAR-Fusion, including the human reference genome, gene structure annotations, and STAR genome index), FusionInspector constructs fusion contigs by extracting the genomic sequences for each geneA and geneB, and concatenating each geneA and geneB pair into a single contig in collinear transcribed orientation. Gene structure annotations for fusion genes are similarly restructured to match the position and orientation of the corresponding genes in the fusion contigs. By default, long introns are shrunk to 1 kb in length by removing central regions of intron sequences, reducing the alignment search space and simplifying downstream visualizations.

RNA-seq reads are aligned to the fusion contigs along with the whole reference genome by running STAR with both inputs, including the pre-indexed whole genome and a fasta file containing the fusion contigs. STAR first loads the whole reference genome index into RAM, then builds an index for the fusion contigs, and incorporates the fusion contig index into the whole genome index. Only those reads that align concordantly to the fusion contigs, while considering all alignments to the combined targets, are reported. Note, that in the fusion context, all fusion-supporting reads are aligned concordantly, but will align partially to one gene and partially to the adjacent gene. This functionality was implemented in STAR since version 2.5.0a to support FusionInspector functionality. STAR-Fusion directly executes STAR to align reads like so " STAR --runThreadN 4 --genomeDir ctat_genome_lib_build_dir/ref_genome.fa.star.idx --outSAMtype BAM SortedByCoordinate --twopassMode Basic --alignSJDBoverhangMin 10 --genomeSuffixLengthMax 10000 --limitBAMsortRAM 47271261705 --alignInsertionFlush Right --alignMatesGapMax 100000 --alignIntronMax 100000 --readFilesIn reads_1.fastq.gz reads_2.fastq.gz --genomeFastaFiles finspector.fa --outSAMfilter KeepAllAddedReferences --sjdbGTFfile finspector.gtf --alignSJstitchMismatchNmax 5 -1 5 5 --scoreGapNoncan −6 --readFilesCommand 'gunzip -c' ", where 'finspector.fa' and 'finspector.gtf' correspond to the fusion contigs sequence and structure annotation files.

FusionInspector examines the aligned reads output by STAR and identifies read alignments supporting fusions between gene pairs represented by the fusion contigs. Candidate fusion breakpoints are identified by split read alignments having partial alignments that anchor to exons of the neighboring fusion genes. Spanning fragments are identified as paired-end reads having each read mapping entirely on opposite sides of the breakpoint. Alignments must meet minimum criteria to be counted as evidence, requiring at least 98% sequence identity and no more than 10 bases unaligned at their ends (soft- or hard-clipped bases). For split reads, at least 10 bases must align adjacent to each breakpoint (anchor), and each anchor region must have sufficient sequence complexity, requiring entropy ≥ 1.2. For spanning fragments, each paired-end read sequence must have sufficient complexity, requiring entropy ≥ 1.2. Preliminary fusion predictions are defined based on candidate fusion breakpoints and sets of compatible spanning fragments. RNA-seq fragments that span a candidate breakpoint but support transcription from an unfused partner gene are captured, stored as counter-evidence, and used to compute the partner gene counter FFPM and fusion allelic ratio.

There is often evidence for multiple fusion variants, and while the split reads are unique to and define each breakpoint, the spanning fragments are often compatible with multiple breakpoints and assigned ambiguously. We implemented an expectation maximization (EM) algorithm based on that described in kallisto^83^ to fractionally assign RNA-seq evidence fragments to fusion variants according to maximum likelihood. Fusion expression values (FFPM) are then computed based on estimated RNA-seq fragment counts resulting from the EM.

Fusion candidates are then filtered according to defined minimum evidence requirement, with defaults set as requiring at least one split read to define the junction breakpoint, and at least 25 aligned bases supported by at least one read on both sides of the fusion breakpoint (termed ‘long double anchor support’ or ‘LDAS’). If the breakpoint involves non-consensus dinucleotide splice sites, then at least three split reads are required to support the breakpoint. A final filter of fusion predictions to exclude those containing overly promiscuous fusion partners (maximum 10) or those involving paralogs of more dominantly supported fusions is applied identically as previously described.^84^

Optionally, Trinity *de novo* assembly^75^^,^^76^ is integrated to *de novo* reconstruct candidate fusion transcripts based on reads aligning to the fusion contigs. When employed, Trinity-reconstructed fusion transcripts are identified in the final FusionInspector report, and the assembled transcripts are available for further study. In addition, FusionInspector integrates IGV-reports^85^ to generate an interactive web-based summary (and fully self-contained html file) of predicted fusions coupled to a web-based interactive genome viewer to examine the read alignments found as evidence for the fusions (Figure S1B).

#### Applications of FusionInspector to TCGA, GTEx, TARGET, and cell lines

FusionInspector was run on TCGA v11, GTEx v8, and TARGET via Terra/AnVIL,^86^ as outlined in our analysis roadmap (Figure S1A). First, FusionInspector v2.4.0 was used to reexamine a subset of 628 TCGA and 530 GTEx samples identified as containing instances of recurrent STAR-Fusion (v1.7) predictions. Candidate samples were identified based on individual fusions (a) having minimum 0.1 FFPM, (b) found in tissue types with at least three occurrences, and either (c) comprising at least 10% of samples of that tissue type, or (d) containing a recurrent COSMIC fusion (ie. (a & b) & (c |d)). Samples were then greedily selected to maximize recurrent fusion content while minimizing numbers of selected samples, retaining up to 10 samples per fusion. These samples and all fusions predicted among these samples (not restricted to those recurrent fusions leveraged as sample selection criteria) were reexamined by executing the current STAR-Fusion (v1.9.1) including FusionInspector (v2.4.0) as a post-process like so: "STAR-Fusion --left_fq ${sample_name}_1.fastq --right_fq ${sample_name}_2.fastq --CPU 16 --genome_lib_dir ctat_genome_lib_build_dir --output_dir ${sample_name} --FusionInspector validate --no_annotation_filter --min_FFPM 0.0 ″ leveraging companion CTAT genome library "GRCh38_gencode_v22_CTAT_lib_Apr032020". The FusionInspector abridged outputs were consolidated and presented as Table S3. These fusions were subsequently subject to Leiden clustering^87^ (see *Fusion Clustering and Class Prediction* section below).

Second, FusionInspector was run in fusion screening mode to explore instances of defined COSMIC-peak-enriched fusions (Leiden cluster 4 (C4) of the 61 fusion clusters found to be heavily enriched for COSMIC fusions). There were 231 instances of C4 fusions selected according to the following criteria: found in at least 3 samples, at least one fusion occurrence found clustered to C4, and at least 30% of occurrences found localized to clusters containing at least two known COSMIC fusion occurrences. These were further supplemented with five recurrent COSMIC fusions that are not members of C4 (ERC1::RET, SLC34A2::ROS1, SS18::SSX1, SS18::SSX2, and VTI1A::TCF7L2), to a total of 236 fusion gene pairs (Table S4). The 236 fusion gene targets were provided as input to FusionInspector for screening 2,764 TCGA and 1,009 GTEx samples, each with the same list of 236 candidates. These samples were selected based on having a STAR-Fusion predicted occurrence of at least one of these fusions (from Table S2) and selecting a maximum of 50 samples per-fusion gene-pairing (with samples sometimes containing multiple fusion types), except for pancreatic and prostate cancer (TCGA) and normal pancreas tissue (GTEx) for which all samples were selected as targets. FusionInspector was executed like so: "FusionInspector --fusions $Table_S4_fusions --genome_lib_dir ctat_genome_lib_build_dir -O ${sample_name} --left_fq ${sample_name}_1.fastq --right_fq ${sample_name}_2.fastq --out_prefix ${sample_name} --vis" leveraging companion CTAT genome library "GRCh38_gencode_v22_CTAT_lib_Apr032020", and results for screening of these samples are provided in Table S5. TARGET RNA-seq samples were processed as above first leveraging STAR-Fusion-v1.10.0 for initial fusion candidate identification followed by FusionInspector-v2.4.0 for in silico fusion validation.

Using RNA-seq data for breast cancer cell lines BT474, MCF7, KPL4, and SKBR3 as in,^11^ FusionInspector-v2.4.0 was run on each sample with a targeted list of 52 experimentally validated fusions (Table S1).

#### Fusion transcript clustering and attribute class prediction

All 53,240 fusion variants surveyed by FusionInspector from our initial subset of TCGA and GTEx samples were clustered according to sequence and expression characteristics. Microhomologies defined as exact *k*-mers with *k* = 10 were identified between candidate fusion gene pairs as represented in the FusionInspector-constructed fusion contigs (with introns shrunk to a max of 1 kb each for simpler visualizations). The Euclidean distance of each candidate fusion breakpoint to the nearest site of microhomology was determined in the FusionInspector fusion contig coordinate system. Attributes of interest for clustering fusions were: (1) the fusion expression level (FFPM), (2,3) partner gene fusion allelic ratios (5′-FAR and 3′-FAR), (4,5) the left and right unfused partner gene expression levels expressed as 5′- and 3'-counter-FFPM and computed based on the number of counter-reads observed as aligned at each corresponding gene breakpoint site, (6,7) indicators for consensus dinucleotides and agreement with reference gene structure exon boundaries at the fusion breakpoints, and (8) the number of microhomologies and (9) distance of the breakpoint to the nearest microhomology. These numerical values were centered and scaled to Z-scores, truncated within the interval [-2,2] to remove outliers, and then rescaled so each attribute numerical vector would fill the interval [-2,2] simplifying our evaluation of metrics using a consistent low-to-high range for each attribute type.

We calculated the distance between fusions based on vectors with these values, constructed a *k*-nearest-neighbor graph (*k*=50) of fusions, and clustered the graph by Leiden clustering^87^ (resolution parameters set as 3 for TCGA & GTEx, and 2 for TARGET). The impact of the resolution parameter on clustering and COSMIC fusion enrichment was examined (Figure S4), and the parameter with sufficiently granular set of clusters was selected for further analysis. Clusters were manually reviewed and grouped and annotated according to median cluster attributes, with cluster annotation term assignments as "COSMIC-like" if predicted as cluster C4, and categories "High FAR" and "Microhomology RT-induced artifact" to reflect likely bioinformatic or reverse-transcription related artifacts (as labeled in Figure 5B).

A random forest classifier was built to predict Leiden cluster membership based on each fusion variant’s scaled feature attributes. The classifier was constructed by randomly selecting a maximum of 300 fusions (median cluster size) from each cluster, and leveraging 2/3 of fusions for training and 1/3 for testing, all performed using Ranger.^88^ Fusions predicted to be assigned to any cluster noted earlier with a fusion cluster annotation (*e*.*g*., "COSMIC-like") are assigned a prediction according to that fusion cluster annotation term. Such fusion attribute cluster predictions are now incorporated into FusionInspector (v2.8.0).

### Quantification and statistical analysis

Statistical tests were performed using R as indicated, with P-values adjusted by Benjamini-Hochberg procedure to control the false discovery rate. Wilcoxon rank-sum test was restricted to comparisons involving at least three members of each set. All statistical analyses performed are included within the Supplementary Code (key resources table).

## Data Availability

• This paper analyzes existing, publicly available data. These accession numbers for the datasets are listed in the key resources table. • All original code has been deposited at GitHub and Zenodo and is publicly available as of the date of publication. URLs and DOIs are listed in the key resources table. • Any additional information required to reanalyze the data reported in this paper is available from the lead contact upon request.

## Supplemental information

Document S1. Figures S1–S10

Table S1. Fusions predicted among breast cancer cell lines, related to Figure 2

Table S2. STAR-Fusion predictions for TCGA and GTEx, related to Figure 4

Table S3. FusionInspector results for selected TCGA and GTEx samples, related to Figure 5

Table S4. FusionInspector results for the targeted C4+ fusions, related to Figure 6

Table S5. FusionInspector results for additional COSMIC fusions, related to Figure S8A

Table S6. STAR-Fusion predictions and corresponding FusionInspector characterizations for TARGET pediatric cancer RNA-seq samples, related to Figures 7 and S9

Document S2. Article plus supplemental information
