# Supplementary material for: Targeted in silico characterization of fusion transcripts in tumor and normal tissues via FusionInspector
Source: Cell Rep Methods. 2023 May 8;3(5):100467. doi: 10.1016/j.crmeth.2023.100467 (PMC10261907; doi:10.1016/j.crmeth.2023.100467)
Supplement: Document S1. Figures S1–S10 [file mmc1.pdf]

Cell Reports Methods, Volume *3*

Supplemental information

Targeted *in silico* characterization of fusion transcripts in tumor and normal tissues via FusionInspector

Brian J. Haas, Alexander Dobin, Mahmoud Ghandi, Anne Van Arsdale, Timothy Tickle, James T. Robinson, Riaz Gillani, Simon Kasif, and Aviv Regev

List of Supplemental Figures

Figure S1: FusionInspector Development, Visualization, and Application

Figure S2: Fusion Transcript COL1A1--FN1 Identified Recurrently Among Cancer Associated Fibroblast Cell Lines in the Cancer Cell Line Encyclopedia is Likely Artifactual.

Figure S3: Higher Number of Fusion Predictions in Tumor vs. Matched Normal Samples in TCGA but not GTEx.

Figure S4. Number and Sizes of Fusion Clusters vs. Leiden Resolution Setting.

Figure S5: UMAP Ordination of Fusions Painted According to Scaled Fusion Feature Attributes and C4 COSMIC Fusion Allelic Ratios.

Figure S6. Properties of Recurrent C4 and COSMIC Fusions, Ranked by Tumor/Normal Sample Occurrence Ratio.

Figure S7. Fusion Cluster Prediction Accuracy Assessment

Figure S8. Characteristics of Select Fusion Types.

Figure S9: FusionInspector Exploration of TARGET Pediatric Cancers.

Figure S10: FSIP1::RP11-624L4.1 Fusion Potentially Relevant to Breast Cancer.

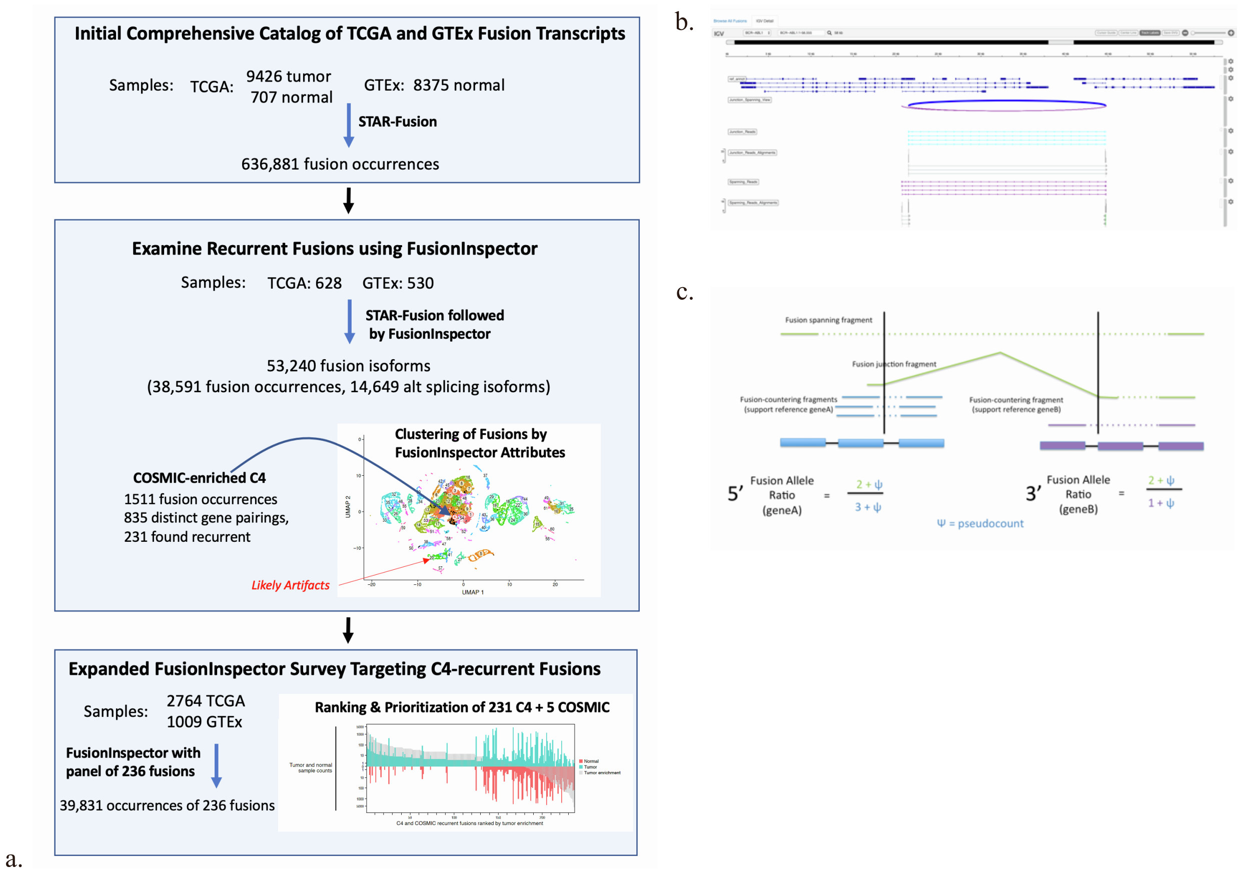

Figure S1. FusionInspector Development, Visualization, and Application. (a) Roadmap for our Intertwined Development and Application of FusionInspector. From an initial comprehensive catalog of fusion transcripts predicted from TCGA and GTEx (**top**), we selected a subset of samples representative of recurrent fusions, which we further investigated using FusionInspector (**center**). Clustering of inspected fusion transcript isoforms based on FusionInspector computed attributes coupled with annotation of known cancer fusions yielded our discovery of COSMIC-fusion enriched cluster C4. Certain other clusters had features consistent with likely artifacts. Using fusion cluster assignments, we built a random forest classifier to predict new fusion instances according to cluster labels based on their FusionInspector attributes, ultimately yielding predictions of new fusion isoforms as COSMIC-like, artifact-like, or other fusion cluster type. To gain further insights into additional COSMIC-like fusions that might be prioritized for further study, we targeted FusionInspector to numerous additional predicted instances of these C4 fusions across TCGA and GTEx samples (**bottom**). We examined predicted fusion type classifications as predominantly COSMIC-like, artifact-like, or other, coupled with functional impacts on coding sequences, prevalence of occurrence across normal and tumor samples, and we prioritized fusions accordingly, demonstrating the utility of FusionInspector for in silico characterization and prioritization of predicted fusion transcripts while identifying novel fusion isoforms of potential relevance to tumor or normal biology. **(b) FusionInspector Visualization Leveraging IGV-reports**. Fusion evidence reads are shown for fusion BCR::ABL1 aligned to the FusionInspector fusion contig in comparison to the reference gene structures. The FusionInspector igv-reports are stand-alone html application files provided as and output file and convenient for rapid interactive data exploration in any computing framework including cloud-computing architectures such as Terra, or popular platforms such as Galaxy. **(c). Computing 5' and 3' Fusion Allelic Ratios (FAR).** RNA-seq reads supporting the fusion (green) are compared to the RNA-seq reads supporting the unfused partner transcripts (5’: blue; 3’: red) at the breakpoint to compute 5’- and 3’-gene fusion allelic ratios. In practice, we use pseudocount=1. Relates to figs 1, 5, 6.

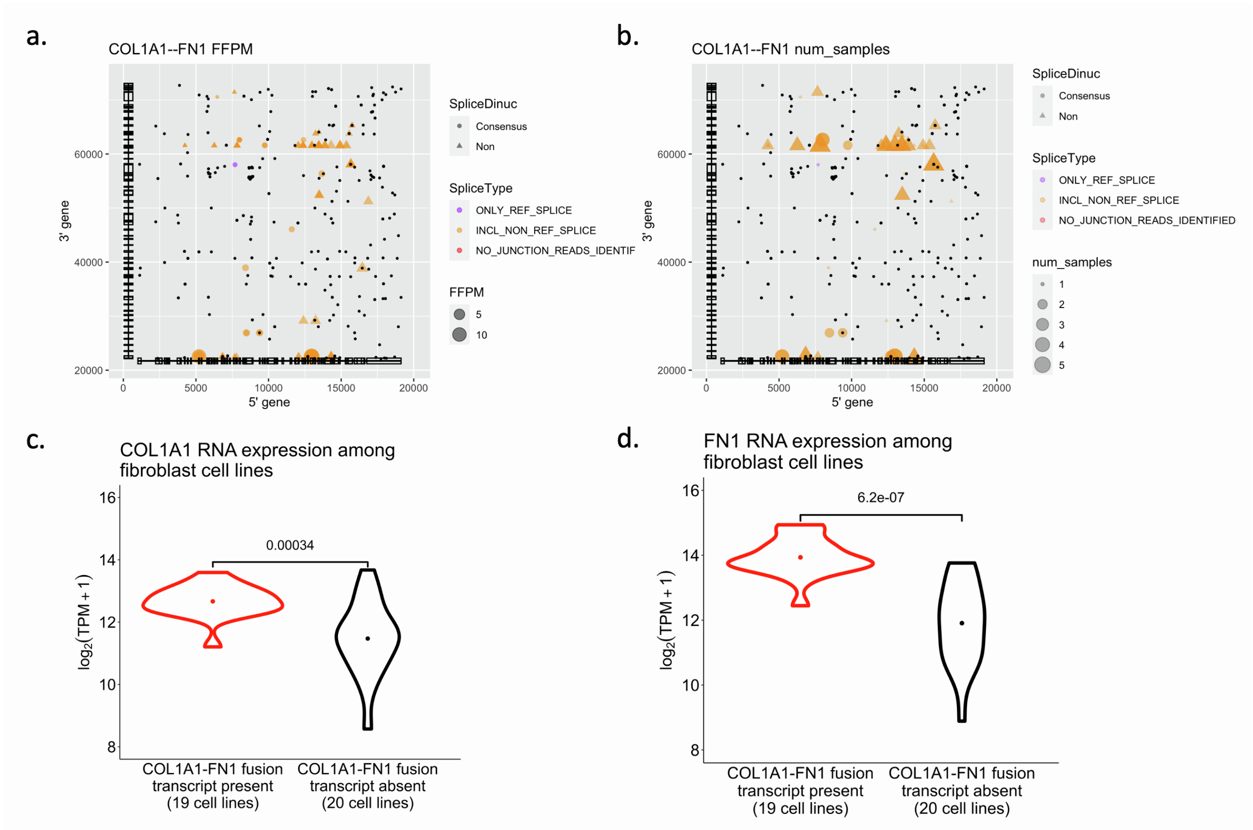

Figure S2: Fusion Transcript COL1A1--FN1 Identified Recurrently Among Cancer Associated Fibroblast Cell Lines in the Cancer Cell Line Encyclopedia is Likely Artifactual. (**a,b**) Isoform expression levels for putative fusions (**a,** dot size) or number of samples (**b**, dot size), splice type (dot color) and splice junction dinucleotide (dot shape) at each fusion breakpoint position involving the 5' (x axis) and 3' (y axis) partners of COL1A1::FN1 in five fibroblast cell lines with highest COL1A1::FN1 fusion read support (HS600T_FIBROBLAST, HS688AT_FIBROBLAST, HS739T_FIBROBLAST, HS819T_FIBROBLAST, and HS822T_FIBROBLAST). Black dots: positions of microhomology (10 base exact match). Structures of collapsed isoforms for fusion partner genes are drawn along each axis. (**c,d**) Distribution of (c) COL1A1 or (d) FN1 expression levels (x axis) in fibroblast cell lines with (red) or without (black) a detected COL1A1::FN1 fusion. Expression data are derived from DepMap (https://depmap.org/portal/download/) release 19q2. Because most of the reads contributing to the robust FFPM are spanning fragments, most breakpoints occur at non-canonical dinucleotide splice sites, and there is significant microhomology detected at these breakpoints, COL1A1::FN1 is likely an RT-PCR artifact resulting from increased expression of partner genes, as opposed to the molecular lesion. Relates to Figures 1, 3.

a.

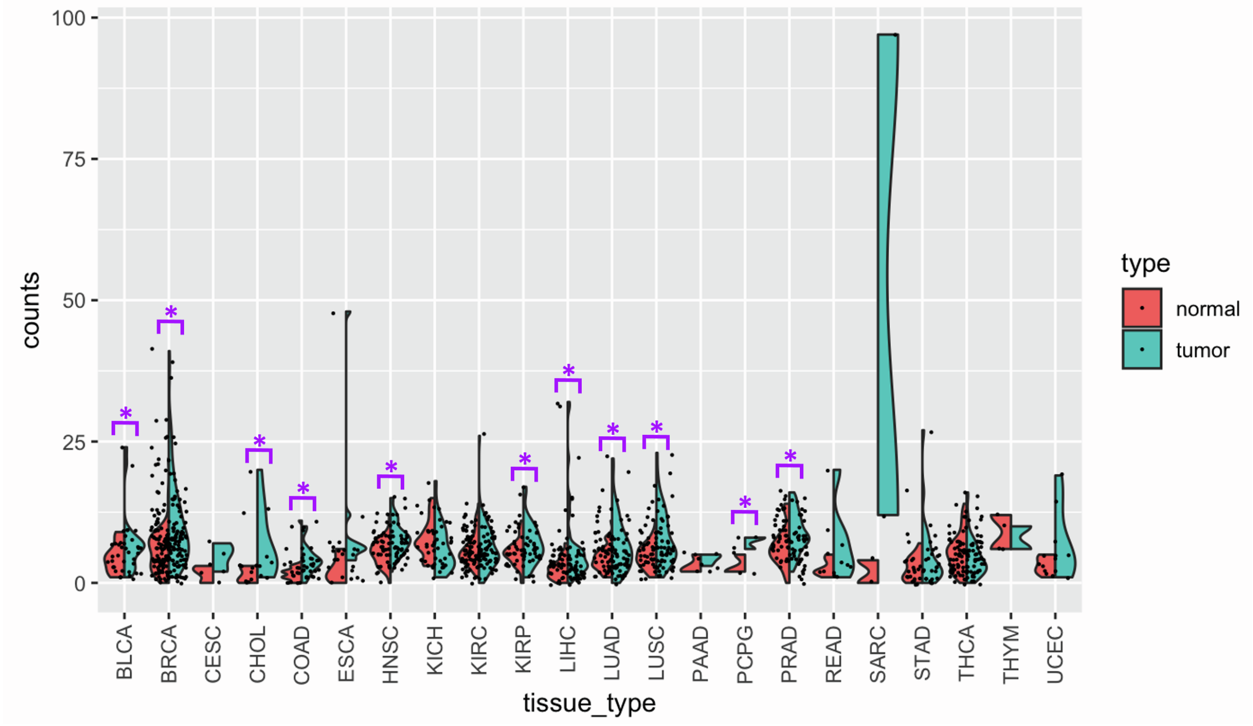

b.

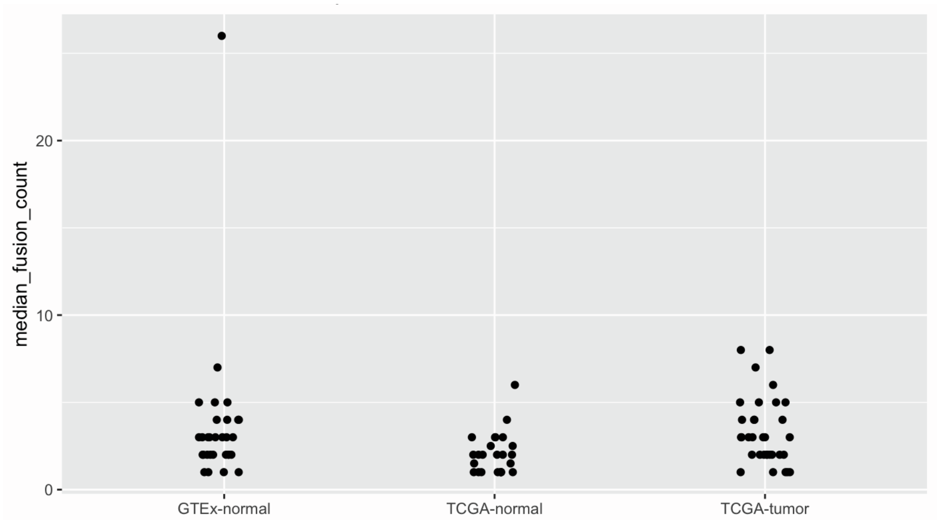

Figure S3: Higher Number of Fusion Predictions in Tumor vs. Matched Normal Samples in TCGA but not GTEx. **(a)** Higher number of fusions in tumors vs. matched normal samples in multiple tumor types in TCGA. Distribution of number of fusions (y axis) in each tumor (blue) and matched normal (red) tissue type (x axis) from TCGA, for those fusions where there is at least one fusion in either tumor or matched normal sample at 0.1 FFPM threshold. * Benjamini-Hochberg FDR < 0.05, one-sided paired t-test. **(b)** Similar number (p-value=0.6, t-test) of leniently predicted fusions in TCGA and GTEx. Median number of fusions (y axis) predicted per tumor or normal tissue type (dots) (x axis) after filtering for a minimum of 0.1 FFPM and removing likely readthrough transcripts involving co-linear neighboring genes within 100kb. with typically four to five fusions predicted per normal or tumor tissue type. In normal tissues, pancreas is an outlier (median of 27 fusions per sample). Fusions were identified with lenient evidence requirements to allow sensitive detection: a minimum of only two supporting reads with at least one defining the fusion breakpoint and disabling annotation-based filters (**Methods**). Relates to Figure 4.

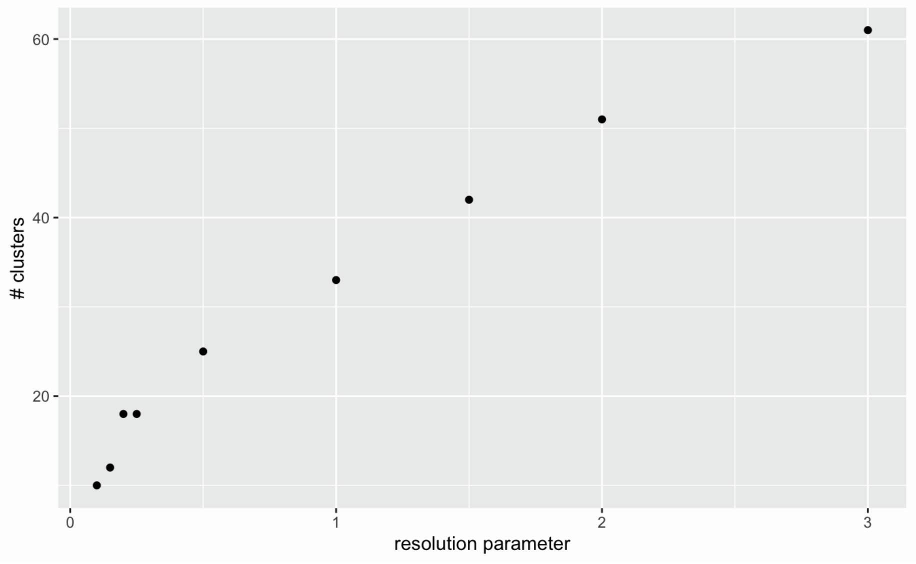

a.

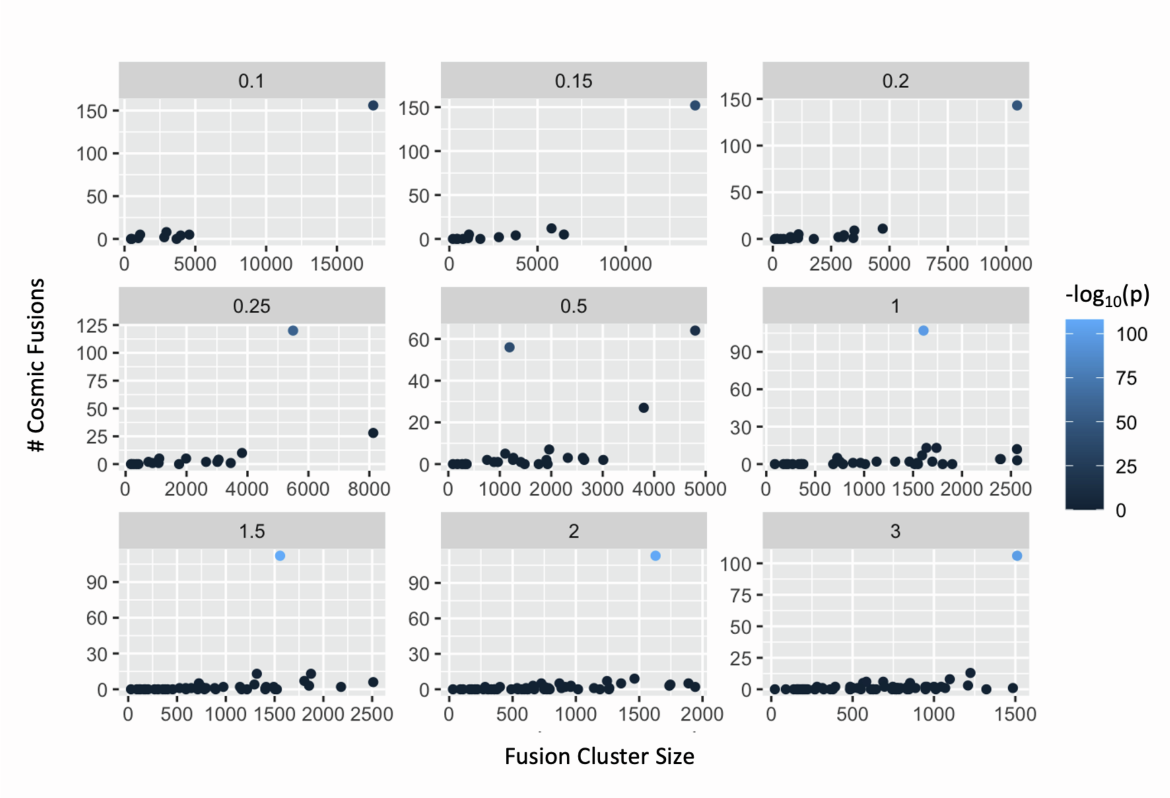

b.

Figure S4. Number and Sizes of Fusion Clusters *vs.* Leiden Resolution Setting. (a) Increasing number of clusters at higher resolution parameters. Number of clusters (*y* axis) at each Leiden resolution parameter value (*x* axis). **(b)** Cluster size (x axis) and the number of COSMIC fusions (y axis) that are members of the cluster for each cluster (dot). Color: $-\log_{10}$(P-value) of enrichment for COSMIC fusion membership (Fisher's exact test, one-sided). Relates to Figure 5.

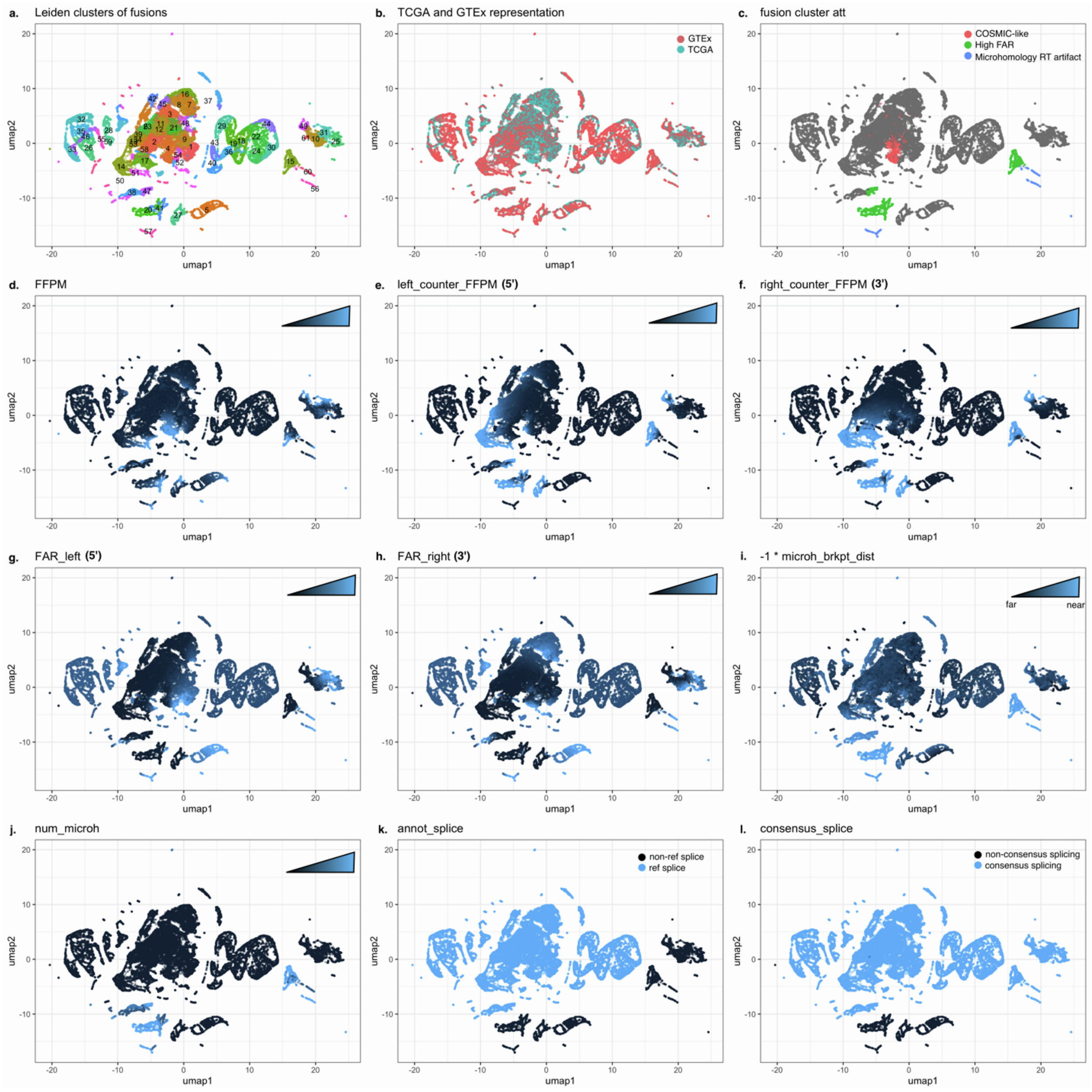

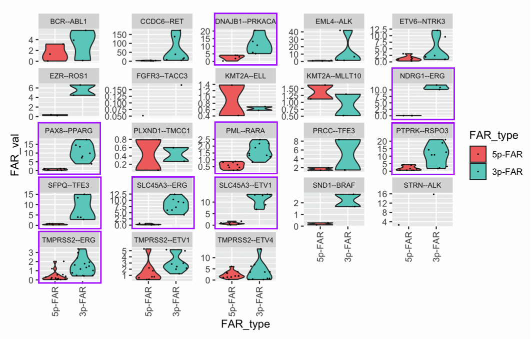

m.

Figure S5: UMAP Ordination of Fusions Painted According to Scaled Fusion Feature Attributes and C4 COSMIC Fusion Allelic Ratios. Fusion variants are painted in the UMAP ordination according to **(a)** Leiden

fusion cluster membership, **(b)** TCGA or GTEx sample occurrence, **(c)** annotated fusion cluster attribute based on feature sequence and expression attributes, **(d)** FFPM fusion expression estimate, **(e)** 5' counter-FFPM, **(f)** 3' counter-FFPM, **(g)** 5' FAR, **(h)** 3' FAR, **(i)** distance of fusion transcript breakpoint to nearest site of microhomology, **(j)** number of microhomologies observed between fusion partner genes as observed in the fusion contig context, **(k)** reference gene structure annotation splice breakpoint indication, and **(l)** consensus dinucleotide splice agreement. See **Supplementary Code** (DOI: 10.5281/zenodo.7791682) for implementation details. **(m) Low 5' vs. 3' FAR for COSMIC Fusions in C4.** Distribution of fusion allele ratio (FAR, y axis) for the 5' (red) and 3' (blue) FAR for 23 representative COSMIC fusions in Cluster C4. Purple: Benjamini-Hochberg FDR < 0.05, one-sided t-test requiring a minimum of 3 samples. Relates to Figure 5.

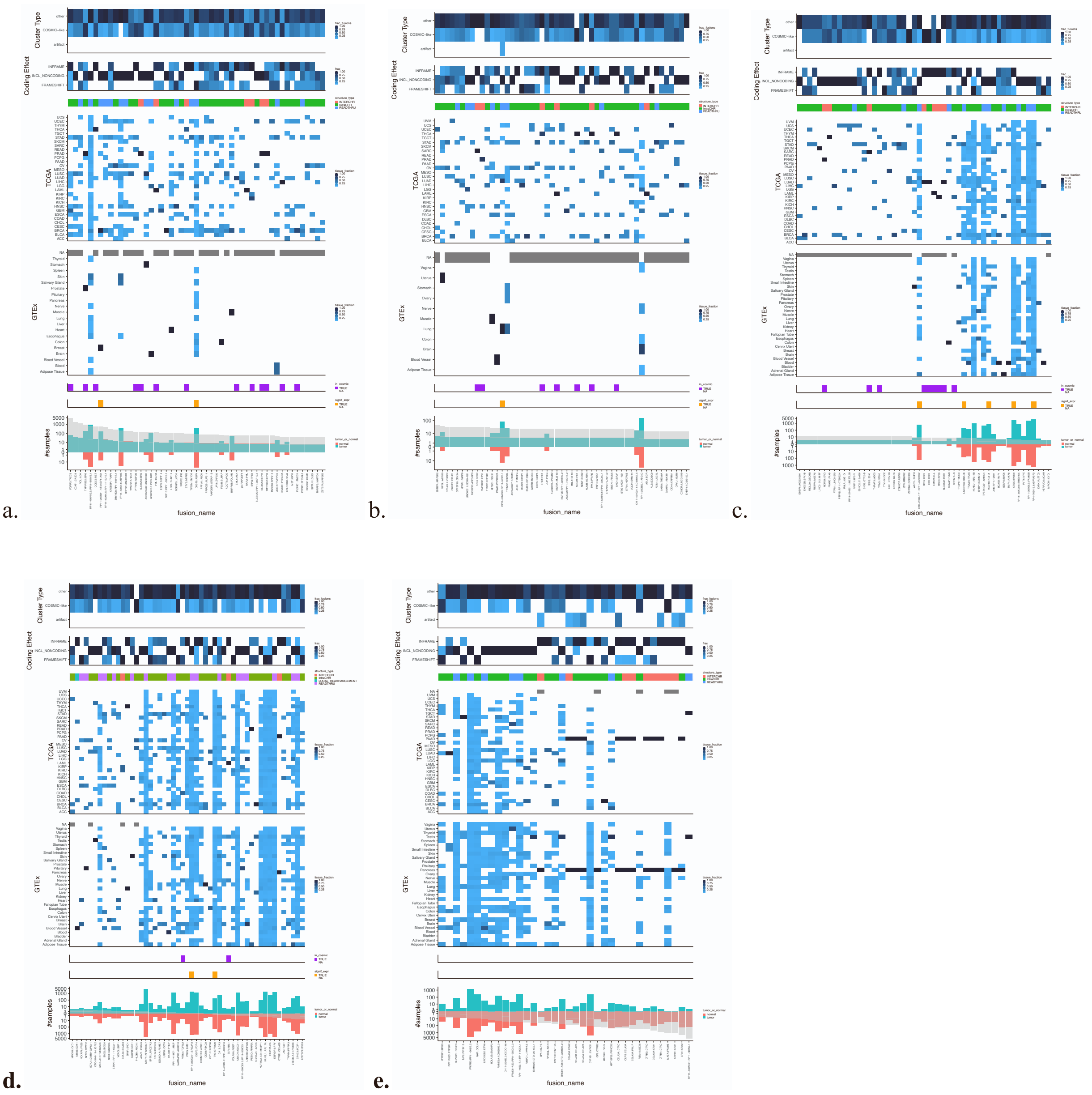

Figure S6. Properties of Recurrent C4 and COSMIC Fusions, Ranked by Tumor/Normal Sample Occurrence Ratio. 236 selected COSMIC-peak-enriched (C4) and additional COSMIC fusions (columns / x axis), rank ordered by tumor enrichment and shown in ~50 fusion increments **(a-e)** with fraction of the instances of each fusion in each category based on predicted Leiden cluster labels (top panel 3 rows) or corresponding to presumed impact on coding sequence (top panel, bottom rows); fusion structure type based on the fusion partner's chromosomal location (second from top); fraction of instances that is in each tumor or tissue type in TCGA and GTEx (third from top, rows); presence in COSMIC (third from bottom, purple), significantly higher expression in tumors *vs*. normal tissues (second from bottom**,** Wilcoxon rank sum test applied to FFPM requiring a minimum of 3 samples for each tumor and normal, Benjamini Hochberg FDR < 0.05 and median tumor FFPM > median normal FFPM, orange), number of tumor (seagreen) or normal (light red) samples (bottom**,** y axis) predicted by STAR-Fusion to contain the fusion, rank ordered by tumor enrichment (bottom, x axis, **Methods**, gray). Zoom on figure to access details. Relates to Figure 6.

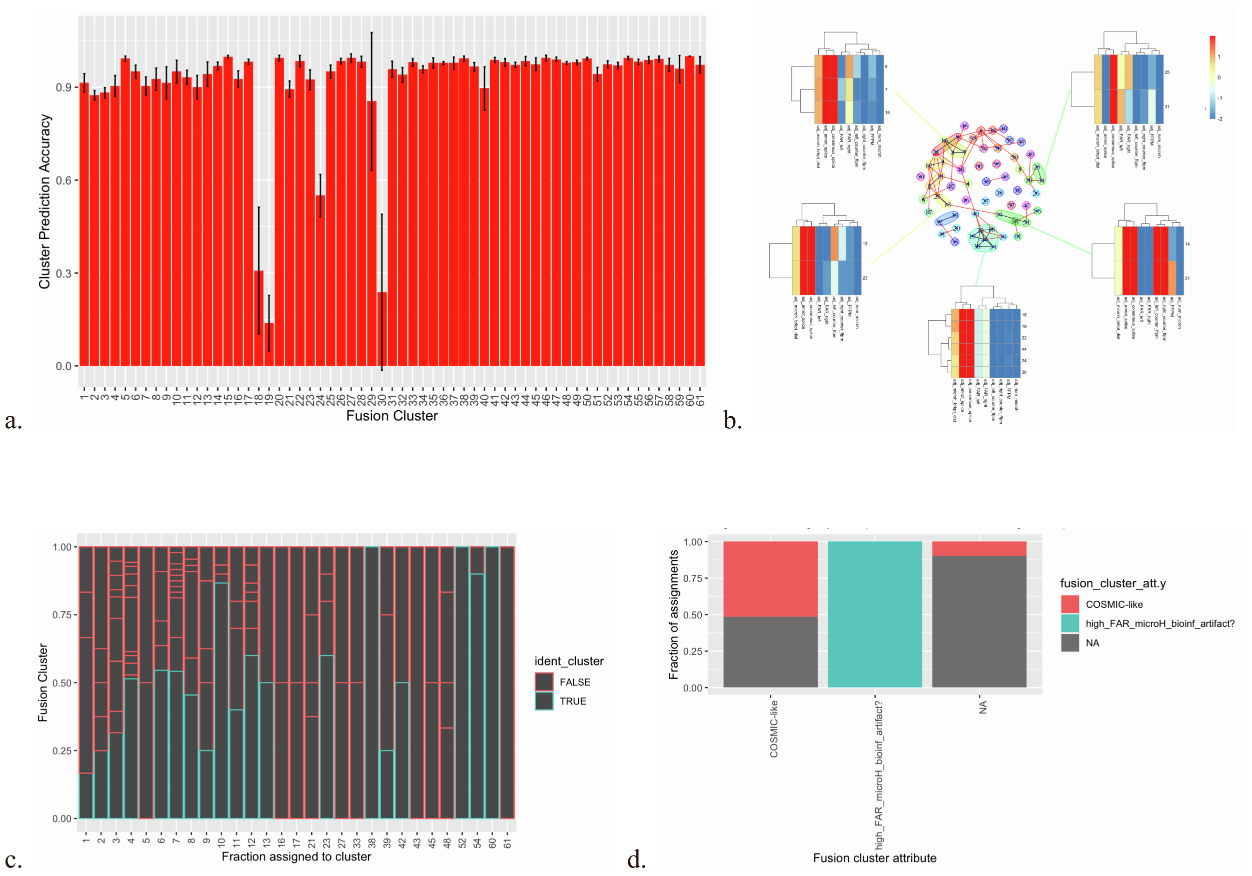

Figure S7. Fusion Cluster Prediction Accuracy Assessment. (a,b) Prediction Accuracy for the Random Forest Classifier. **(a)** Mean prediction accuracy for cluster prediction assignment from 5-fold cross-validation. Error bars show standard deviation above and below the mean. **(b)** Incorrect cluster predictions tend to correspond to clusters having highly similar features. At center is a graph where fusion cluster nodes are vertices and edges are drawn between known and predicted cluster assignments, weighted by the fraction of mis-predictions, followed by clustering. Heatmaps of scaled fusion attribute values are shown for clusters of nodes involving highest rates of mis-predictions, indicating that mis-predicted fusions tend to be assigned to fusion clusters having similar sequence and expression characteristics. See **Supplementary Code** (DOI: 10.5281/zenodo.7791682) for implementation details. (c,d) **Evaluation of Fusion Cluster Predictions for Biological Replicates in TCGA. (c)** Fusion variant cluster predictions in one sample replicate were compared to each other replicate found with the same fusion variant for 20 TCGA participants. All pairs of replicate cluster predictions were tallied, and for a given fusion cluster (x-axis), the fraction of all replicate fusion cluster predictions are plotted (y-axis). Fractions of identical cluster predictions (‘ident_cluster’) between replicates are outlined in blue and disagreeing are outlined in red. For example, more than half of C4 predictions have replicates consistently assigned as C4. **(d)** Fusion cluster comparisons in (c) are reanalyzed at their predicted fusion cluster annotation, again showing that the C4 COSMIC-like fusions tend to have consistent assignments, and predicted artifacts are consistently assigned as artifacts across matched biological samples. See **Supplementary Code** (DOI: 10.5281/zenodo.7791682) for implementation details. Relates to Figures 5,6.

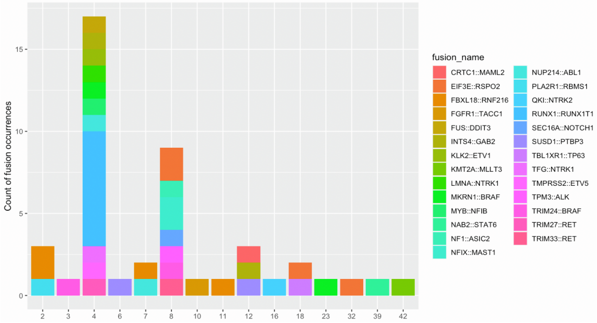

a.

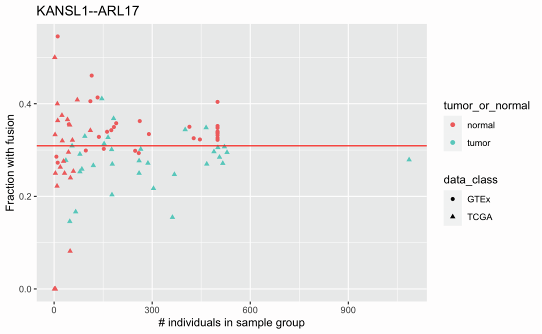

b.

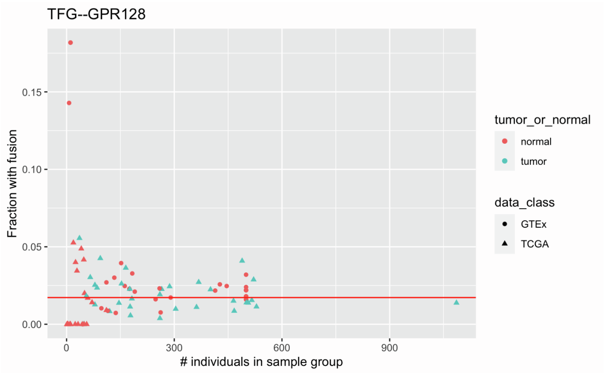

c.

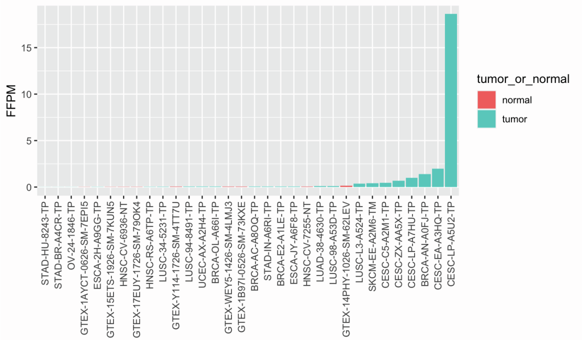

d.

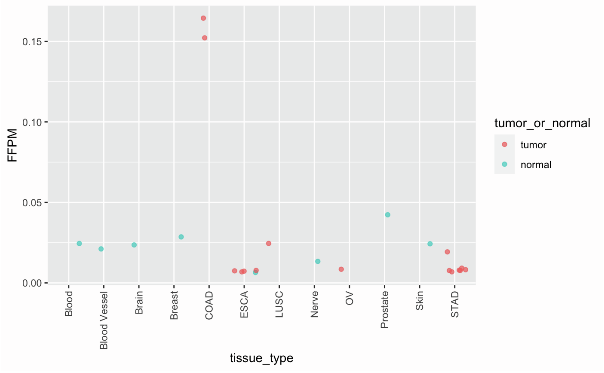

e.

Figure S8. Characteristics of Select Fusion Types. (a) Fusion Cluster Prediction for COSMIC Fusions Subsequently Screened. 45 TCGA and 1 GTEx sample identified via STAR-Fusion as harboring an occurrence of the 27 COSMIC fusions not initially selected for targeted screening were subsequently processed through FusionInspector to examine their predicted fusion cluster assignment. COSMIC-like cluster C4 was found to contain the greatest number of fusion occurrence predictions. None were predicted to artifact-like fusion clusters. **(b) Fraction of TCGA and GTEx Tissue Samples Containing Evidence of Fusion Transcript KANSL1::ARL17.** Each data point corresponds to the fraction of corresponding tissue samples demonstrating evidence of the KANSL1::ARL17 fusion (called as KANSL1::ARL17A or KANSL1::ARL17B). No minimum FFPM threshold applied. **(c) Evidence of Fusion Transcript TFG::GPR128 across TCGA and GTEx**. Fraction of tumor (blue) or normal tissue (red) samples (y axis) with evidence of the TFG::GPR128 fusion transcript, associated with a known germline structural variant estimated with a European population allele frequency of ~2%. No minimum FFPM threshold applied. **(d) Higher PVT1::MYC Fusion Expression in Tumors vs. Normal Samples, Especially in Cervical Cancer.** Expression level (FFPM) of PVT1::MYC fusion in each sample (x axis) in which it is detected. Only the top 11/32 samples have FusionInspector estimated FFPM>0.1, five of which correspond to cervical cancer

(CESC), and corresponds to a hotspot site of HPV insertion in cervical cancer genomes. **(e) The VTI1A::TCF7L2 Fusion is Highly Expressed in Colon Cancer.** Expression level (FFPM) of VTI1A::TCF7L2 fusion in each normal (blue) or tumor (red) sample (*x* axis) in which it is detected (no FFPM threshold applied), ordered by sample type. Relates to Figures 5,6.

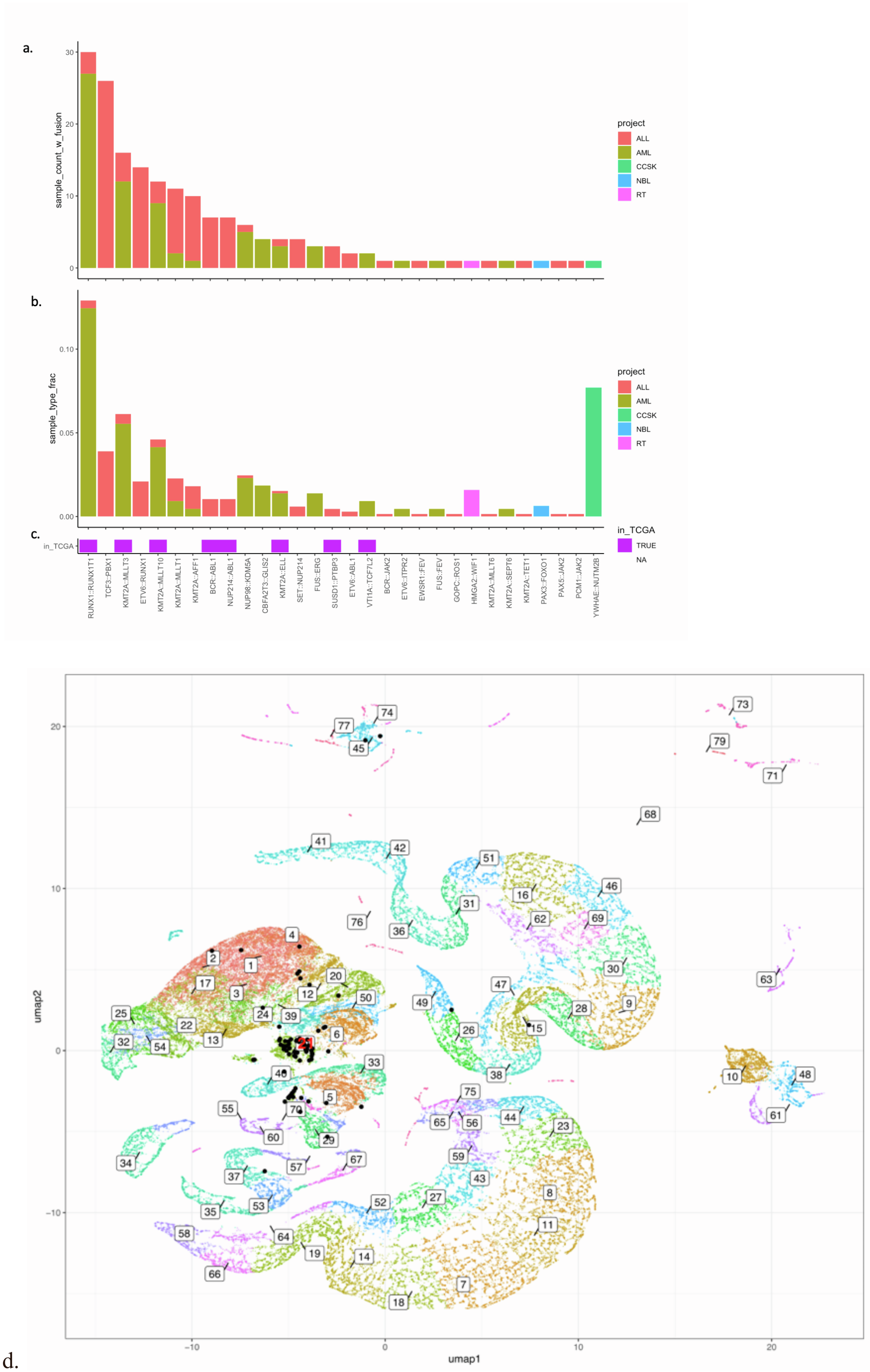

Figure S9: FusionInspector Exploration of TARGET Pediatric Cancers. (a-c) 30 COSMIC Fusions Found in Samples Among TARGET Pediatric Cancers. **(a)** Counts and **(b)** fractions of project participants containing corresponding COSMIC fusion. **(c)** Indicator shown for COSMIC fusions found among TCGA samples. Note that

the PAX3::FOX1 finding in NBL is more typical for rhabdomyosarcoma [S1] and worthy of additional investigation. **(d) UMAP Ordination of TARGET Fusion Variants**. Leiden clusters are labeled, and black dots correspond to COSMIC fusion occurrences. Cluster 21(red label) is found significantly enriched for COSMIC fusion occurrences. All variants were Leiden clustered with Leiden resolution=2. Half of fusion variants (>100k) were randomly selected for the above UMAP visualization. See **Supplementary Code** (DOI: 10.5281/zenodo.7791682) for implementation details. Relates to Figure 7.

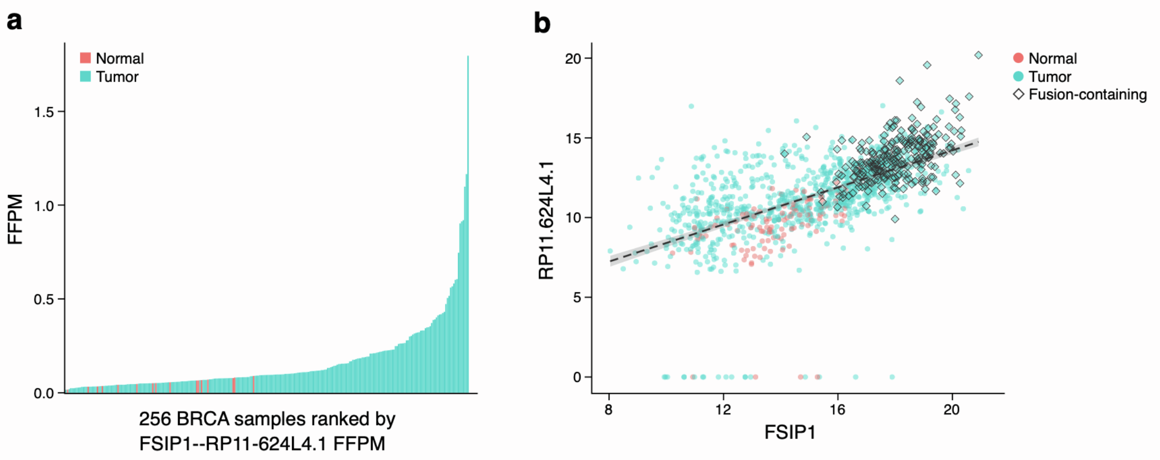

Figure S10: FSIP1::RP11-624L4.1 Fusion Potentially Relevant to Breast Cancer. FSIP1 (fibrous sheath interacting protein 1) was previously identified as a prognostic marker for HER2-positive breast cancers and its high expression is associated with poor patient outcomes [S2]. Fusion partner RP11-624L4.1 is a lncRNA, which is collinear and 170kb downstream from FSIP1, and was recently identified as an oncogene relevant to nasopharyngeal carcinoma [S3]. **(a)** Fusion FSIP1::RP11-624L4.1 was detected in 22% of breast cancer tumors studied (240 of 1,086). While it was also detected in 14 normal breast samples (3 TCGA, 11 GTEx) and two additional samples (prostate and esophagus), its expression was significantly higher in tumors than normal tissue (expression level (FFPM, y axis) of FSIP1--RP11-624L4.1 fusion in tumor (blue) and normal (red) TCGA breast cancer samples, ranked by FSIP1--RP11-624L4.1 expression, Benjamini Hochberg FDR < 0.004, Wilcoxon rank sum test). **(b)** FSIP1 and RP11-624L4.1 expression is positively correlated in both tumor and normal tissues (Pearson $r = 0.6$) and the fusion FSIP1::RP11-624L4.1 is found only among those samples most highly expressing both fusion partners. Expression levels of FSIP1 (x axis) and RP11-624L4.1 (y axis) in each tumor (blue) and normal (red) samples (Pearson r=0.6, p-value < 2.2e-16). Diamonds: samples where the fusion trsanscript is detected. Expression values were $\log_2$ transformed upper-quartile normalized gene FPKM measurements obtained from the Xena platform [S4]. Given the proximity and collinearity of the oncogenic fusion partners, the FSIP1::RP11-624L4.1 fusion transcript likely derives from read-through transcription and cis-splicing. Relates to Figure 5.

References for Supplementary Information

S1. Linardic, C.M., *PAX3-FOXO1 fusion gene in rhabdomyosarcoma.* Cancer Lett, 2008. **270**(1): p. 10-8.

S2. Yan, M., et al., *Over-expression of FSIP1 promotes breast cancer progression and confers resistance to docetaxel via MRP1 stabilization.* Cell Death Dis, 2019. **10**(3): p. 204.

S3. Zhou, L., et al., *lncRNA RP11-624L4.1 Is Associated with Unfavorable Prognosis and Promotes Proliferation via the CDK4/6-Cyclin D1-Rb-E2F1 Pathway in NPC.* Mol Ther Nucleic Acids, 2020. **22**: p. 1025-1039.

S4. Goldman, M.J., et al., *Visualizing and interpreting cancer genomics data via the Xena platform.* Nat Biotechnol, 2020. **38**(6): p. 675-678.
